# Genome-Wide Screen and Validation of Microglia Pro-Inflammatory Mediators in Stroke

**DOI:** 10.14336/AD.2020.0926

**Published:** 2021-06-01

**Authors:** Jianhua Wu, Zhuoze Wu, Aodi He, Tongmei Zhang, Ping Zhang, Jing Jin, Sisi Li, Gaigai Li, Xinyan Li, Shiqi Liang, Lei Pei, Rong Liu, Qing Tian, Ximiao He, Youming Lu, Zhouping Tang, Hao Li

**Affiliations:** ^1^Department of Physiology, School of Basic Medicine and Tongji Medical College, Huazhong University of Science and Technology, Wuhan, China.; ^2^The Institute for Brain Research, Collaborative Innovation Center for Brain Science, Huazhong University of Science and Technology, Wuhan, China.; ^3^Department of Pharmacy, Zhongnan Hospital of Wuhan University, Wuhan, China.; ^4^Department of Neurology, Tongji Hospital, Tongji College of Medicine, Huazhong University of Science and Technology, Wuhan, China.; ^5^Department of Neurobiology, School of Basic Medicine and Tongji Medical College, Huazhong University of Science and Technology, Wuhan, China.; ^6^Department of Pathophysiology, School of Basic Medicine and Tongji Medical College, Huazhong University of Science and Technology, Wuhan, China

**Keywords:** stroke, microglia, pro-inflammatory factor, Hmgb2, Ctss

## Abstract

Stroke activates microglia pro-inflammatory response that not only induces the early neuronal injuries but also causes the secondary brain infarction. Yet, the underlying mechanisms for how microglia become activated in stroke are still unknown. Here, using the next-generation of RNA sequencing we find a total of 778 genes increasingly expressed in brain of stroke mice. Of these, we identified Hmgb2 as a microglia pro-inflammatory mediator by promoting the transcription of Ctss. Inhibition of either Hmgb2 or Ctss blocks microglia pro-inflammatory response and protects against brain damages and improves the neurological functions of stroke mice. This study uncovers Hmgb2 and Ctss as the major microglia inflammatory response mediators in stroke and hence warrants the promising targets for stroke therapies.

Ischemic stroke or brain attack is caused due to disruption of blood flow supplying the brain and is the first leading cause of death in China and the second leading cause of death worldwide [1]. The incidence and mortality of stroke increase with age. As the elderly population is rapidly growing, stroke becomes a common social burden with substantial economic costs [2]. Despite of great advances in understanding the diverse mechanisms of stroke damages, thrombolysis with tissue plasminogen activator (tPA) still is the only clinically effective therapy, but fewer than 5% stroke patients receive effective treatment due to the narrow therapeutic window. Currently, various studies aimed to the ischemic cascade indicated that suppression of inflammation offers potential therapeutic strategies [3].

Stroke activates microglia that induces pro- and anti-inflammatory and immune responses. Under the physiological conditions, microglia produce anti-inflammatory response for neuronal survival and generate a defensive reactions such as phagocytosis for the clearance of harmful substances and apoptotic debris [4]. Microglia could perform different phenotypes like macrophages which is called polarization. Based on the stimulation of pro- and anti-inflammatory cytokines, microglia could polarize into pro- inflammatory (M1 phenotype) and anti-inflammatory phenotype (M2 phenotype)[1]. After stroke onset, there is an M2-to-M1 dynamic shift had been reported that M2 microglia were observed gradually within 7 days after injury, but with the sustained increase of M1 microglia from day 3 to 14 days, M1 microglia began to dominate the peri-infarct region [5]. However, M1 microglia are excessively activated, resulting in a series of pro-inflammatory responses that not only amplify the early tissue damages but also cause the secondary infarct expansion. These studies suggested that the polarized direction of microglia plays critical roles during stroke and inhibition of microglia activation directly seems to be a poor choice. Hence, intervention of microglia pro-inflammatory response while preserving anti-inflammatory functions is considered a promising strategy for the treatment of stroke.

However, lots of targets have been found by micro-array chromatin immunoprecipitation (CHIP) and genome sequencing which varies from apoptosis related genes to inflammatory molecules. But there are still no effective strategies to the excessive inflammatory reaction. So that we hypothesis that proteins involved in the pro-inflammatory response of microglia may be different at different stages of stroke which may be combined and cooperated together, and either of the stage rescued may protect against brain damages after stroke. In order to verify this hypothesis and identify the microglia pro-inflammatory mediators in stroke, ischemic brain tissues isolated 1, 3, 7, 14 or 28 days after operation with sham or stroke were subjected to RNA sequencing (RNA-seq) analysis. The expression of 778 protein coding genes was increased in middle cerebral artery occlusion (MCAO)-treated tissues compared to those in sham-operated tissues. Remarkably, high mobility group box 2 (Hmgb2), tumor necrosis factor (TNF) and interleukin (IL)-11 which were pro-inflammatory factors expressed at the first day after stroke onset; cathepsin S (Ctss) and protein tyrosine phosphatase non-receptor type 18 (PTPN18)which are either peptidases or proteases were increased at 3 days and peaked at 7 days after stroke onset; a total of 190 genes such as complement component 3 (C3) and C6 were increased 14 days after stroke onset and peaked 14 days later. Here, we focused on the inflammatory relative factors. Subsequent functional experiments in this study demonstrated that Hmgb2 was a microglia activator and pro-inflammatory mediator. Hmgb2 bond to promoter region of Ctss and activated Ctss transcription. Inhibition of either Hmgb2 or Ctss blocked microglia pro-inflammatory response and protected against brain damages after stroke. Together, we report that Hmgb2 and Ctss as the major microglia inflammatory response mediators in different stages of stroke and hence warrants the promising targets for stroke therapies.

## METHODS AND MATERIALS

### Animals

Male mice (C57BL/6) at 120?±?5 d old were used, unless otherwise specified. Mice were bred and reared under the same conditions in accordance with institutional guidelines and the Animal Care and Use Committee of the animal core facility at Huazhong University of Science and Technology, Wuhan, China, and housed in groups of 3-5 mice/cage under a 12-h light-dark cycle, with lights on at 8:00 a.m., at a consistent ambient temperature (21?±?1 °C) and humidity (50?±?5%). All behavioral tests were conducted during the light phase of the cycle. To target the studies specifically in microglia, we used the Cx3cr1-Cre mice (Stock No::021160, the Jackson laboratory, Bar Harbor, ME, USA), in which the expression of Cre was induced specifically in microglia by administration of tamoxifen [6].

### Focal cerebral ischemia

Focal cerebral ischemia (stroke) was induced by intraluminal middle cerebral artery occlusion (stroke), as described before [7]. Briefly, a 7/0 surgical nylon monofilament with rounded tip was introduced into the left internal carotid through the external carotid stump and advanced 10-13 mm past the carotid bifurcation. Occlusion was confirmed when blood flow was reduced by at least 80% of the baseline. The filament was left in place for 60 min (occlusion) and then withdrawn (reperfusion). The sham-operated animals were treated identically, except that the middle cerebral artery was not occluded after the neck incision. Each mouse was anesthetized with 2% isoflurane and maintained with 1% isoflurane in an oxygen/air mixture by using a gas anesthesia mask in a stereotaxic frame (Stoelting). The rectal temperature was maintained during surgery at 37 ± 0.5°C with a homeothermic blanket (Harvard Apparatus).

To determine the brain damages, MRI was performed after the reperfusion as described before^12,13^. Briefly, mice were anesthetized with isoflurane 1.5-2% and kept at 37°C, and an MR compatible respiration sensor was used to control the animals and performed on a BIOSPEC BMT 47/40 (Bruker) spectrometer operating a 4.7T, equipped with an 11.2-cm actively shielded gradient system, capable of 200 mT/m gradient strength and 80 μs of rise time. A 7-cm bird cage radiofrequency coil was used for transmission and reception. T2-Weighted Imaging (T2WI) were acquired using a rapid acquisition with relaxation enhancement (RARE) technique, with a repetition time (TR) = 1650 s, RARE factor = 16, and inter echo interval = 10 ms, resulting in an effective echo time (TE) = 80 ms, the field of view (FOV) = 4 × 4 × 4 cm^3^. The acquired matrix size was 128 × 128 × 64. These data were zero-filled to obtain a reconstructed matrix size of 128 × 128 × 128.

**Figure 1.**
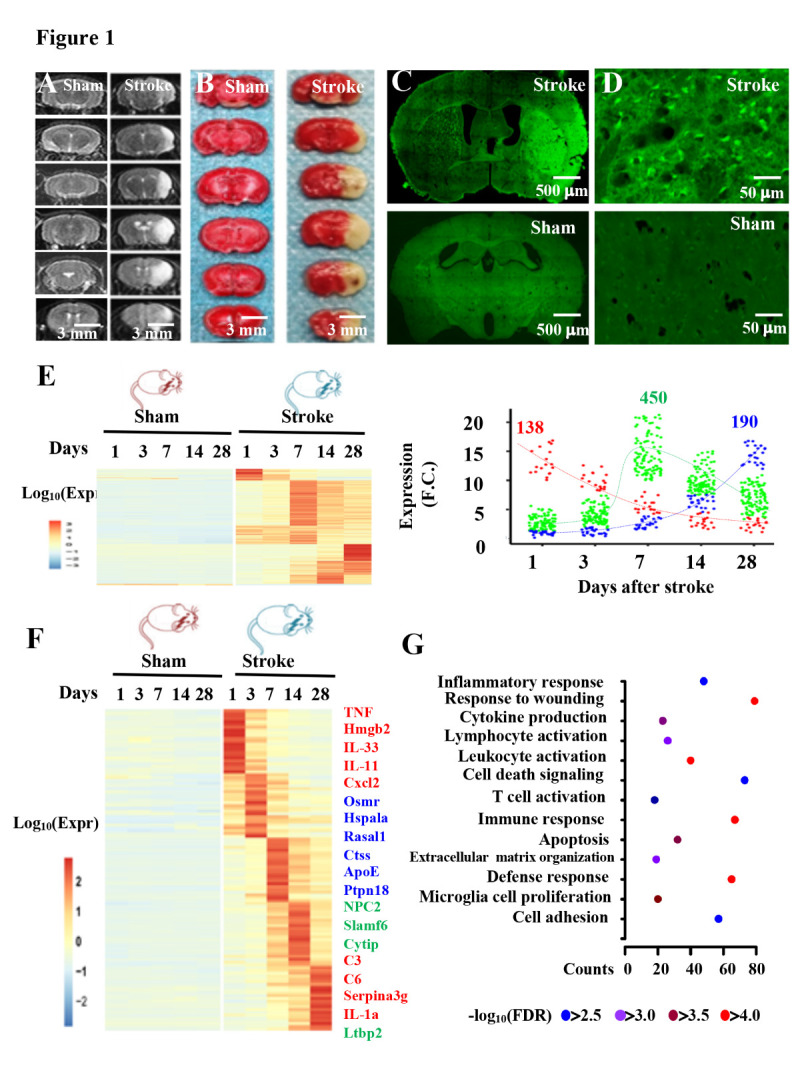
Genome-wide transcriptional alterations in the brain of stroke mice. (A) representative T2 weighted MR images taken 24 hours after operation with shame or stroke. (B) representative images show the brain sections stained with TTC 24 hours after operation with sham or stroke. C, D, low (C) and high (D) magnifications of the images show brain sections stained with FJ at 7 days after operation with sham or stroke. (E) patterns (left) of gene expression in the cortex of mice 1, 3, 7, 14, or 28 days after operation with sham or stroke. A plot (right) shows the DEGs that are classified into three clusters; 138 clsuter one genes (red symbols), 450 cluster two genes (green symbols) and 190 cluster three genes (blue symbols), based on their expression differences across the tine course after stroke onset. (F) the representative DEGs that relate to the microglia pro-inflammatory response (red), proteolitic response and cell death (blue) and neuronal immune response (green) in the cortex of mice 1, 3, 7, 14 or 28 days after operation with stroke, as compared to sham. H, gene ontology analysis of the DEGs in stroke mice.

To determine the brain infarction, TTC staining was performed as described before[7]. Briefly, brain was removed from mice rapidly and frozen at -20°C for 5 min. Coronal slices (7 slices from each mouse) were made at 1 mm from the frontal tips, and sections were immersed in 2% TTC (No: 298-96-4, Sigma) at 37°C for 20 min. The presence or absence of infarctions was determined by examining TTC-stained sections for the areas that did not stain with TTC. To determine cell death, FJ staining was performed as described before[7]. Briefly, the brain tissue slides were immersed for 3 min in 100% ethanol, for 1 min in 70% ethanol, and for 1 min in distilled water and then incubated in a solution containing 0.01% Fluoro-Jade C (AG325, Millipore) and 0.1% acetic acid (1:10) for 30 min on a shaker. After three 10-min washes, the slides were cover-slipped and imaged with a laser-scanning confocal microscope (LSM 510, Carl Zeiss). The number of FJ positive (FJ^+^) cells in each group was counted.

**Figure 2.**
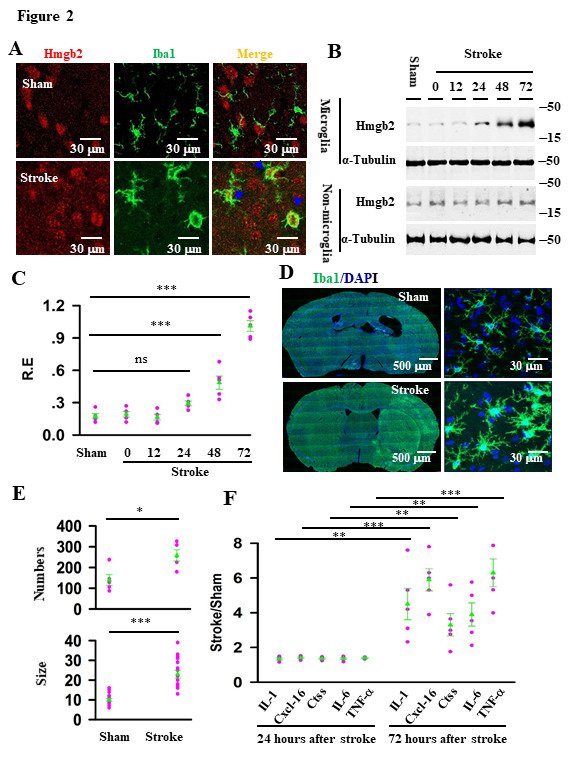
Stroke induces the expression of Hmgb2 in microglia. (A) representative images show the expression of Hmgb2 (red) in micriglia (green) in the cortex at 3 days after operation with sham or stroke, the co-expressed Hmgb2 and Iba1 cells were tagged. (B) the lystaes of microglia and non-microglia fractions from mice at 0, 12, 24, 48, 72 hours after operation with sham or stroke were prepared and blotted with anti-Hmgb2 or anti-α-tubulin, as indicated. (C) Relative expression (R.E) levels (defined by normalizing the band intensities of anti-Hmgb2 to the respective anti-a-tubulin) in the individual mice (circles) and their averages per group (triangles) are plotted. Data are mean ± SEM (0.17 ± 0.024 in sham verse 1.02 ± 0.13 in stroke group, n = 5 mice per group, *F*_(5, 24)_ = 74.61, ns = no significantly differences, ***p < 0.0001, BF ANOVA). (D) representative images show the brain section stained with DAPI (blue) and anti-Iba1 (green) from mice at 3 days after operation with sham or stroke. (E) both the numbers (mean ± SEM, n = 5 mice per group, *p = 0.013 , *t*-tests) and the soma size (mean ± SEM, n = 15 cells/5 mice per group, ***p < 0.001, *t*-tests) of the Iba1-labeted cells in the indivudal mice (circles) and their averages per group (triangles) at 3 days after operation with sham or stroke are plotted. (F) The ratios of the major inflammatory factors (IL-1, Cxcl-16, Ctss, IL-6 and TNF-a ) in stroke versus sham in the individual mice (circles) and their averages per group (triangles) are plotted. Data are mean ± SEM (n = 5 mice per group, **p = 0.0091, 0.0014, 0.0059, ***p < 0.0001, *t*-tests).

To determine the neurological functions, neurological performance was scored (N.S) daily using modified 7-point neurological scales; 0, no observable neurological deficits; 1, flexion of the contralateral torso; 2, circling to the ipsilateral side but normal posture at rest; 3, circling to the ipsilateral side; 4, rolling to the ipsilateral side; 5, leaning to the ipsilateral side at rest; 6, longitudinal spinning; and 7, no spontaneous motor activity/death. Score were ranked from 0 to 7 at an interval of 1.0. Neurological performance was always assessed by the blinded independent investigators.

### RNA-sequencing and bioinformatics analysis

Male mice at 120 ± 5 days old of age were operated with sham or stroke. 1, 3, 7, 14 or 28 days after the operation, the cerebral cortex was isolated and total RNAs were purified using RNeasy Plus Micro kit (74004, Qiagen). A total of 10 groups of mice were used and each group had three replicates. A total of 5 μg RNAs per sample was used as input material for RNA-sequencing library preparations. The RNA quality was evaluated using Experion RNA StdSens Analysis Kit (7007103, BIO-RAD). The sequencing libraries were generated using TruSeq RNA Sample Preparation Kit v2 (RS-122-2001, Illumina) following manufacturer’s instructions. The quality of the library was evaluated using Experion DNA 1K Analysis Kit (7007107.0, BIO-RAD). The index-coded samples were prepared using HiSeq PE Cluster Kit v4 (401-4001, Illumina) in a cBot Cluster Generation System (Illumina). The libraries were sequenced on a HiSeq X Ten sequencer with paired-end reads, 150-cycle sequencing run by Novogene.

We utilized the RNA-sequencing analysis pipeline following the instructions of *hppRNA*, a tool integrating six most popular workflows for RNA-sequencing analysis. We used the Tophat-Cufflinks-Cuffdiff workflow for our analysis. In brief, the known adapters and low-quality regions of reads were trimmed using *Cutadapt* (version 1.9) and the reads with bases of N over 5% were discarded. RNA-sequencing quality was assessed using *FastQC* (version 0.11.5, http://www.bioinformatics.babraham.ac.uk/projects/fastqc/). The RNA-sequencing reads were aligned to the mouse reference genome assembly (mm10) using *Tophat* (version 2.1.1) with default parameters. The mouse genome sequences were downloaded from http://genome.ucsc.edu/cgi-bin/hgGateway?db=mm10. After mapping, the PCR duplicates were removed using *rmdup* in *Samtools*. Subsequently, gene expression was normalized as FPKM (fragments per kilobase of exon per million fragments mapped) by *Cufflinks* (version 2.2.1) using Ensembl’s gene annotation for mm10. The genes with FPKM below 0.5 in all samples were filtered as non-detectable. Finally, the differential expression genes (DEGs) were identified using *Cuffdiff* (version 2.2.1). The DEGs were defined as the ones with fold-change (F.C) more than 2 and *p* < 0.05.

To analyze the clusters of DEGs, we calculated the mapping reads count per gene for each sample at different time points after operation with sham or stroke (Day 1, 3, 7, 14 and 28 after stroke onset) by *edegR*[8]. We identified the new gene sets of DEGs by RNA-sequencing time series analysis using R package *maSigPro* with a Generalized Linear Models (GLM) method. The analysis was performed using default parameters and DEGs were defined as the genes with FDR < 0.05. Finally, all the DEGs were classified into three clusters with K-means (K=4) using *maSigPro* according to the gene expression patterns across the time course after stroke onset.

To analyze gene ontology (GO) and pathway enrichments, we used *enrichGO* in R package *clusterProfiler* (version 3.6.0) to identify the enriched biological process (BP) gene function terms. The significance of enriched GO terms was evaluated by a hyper-geometric test with FDR < 0.05. We also used *enrichKEGG* in R package *clusterProfiler* to identify enriched KEGG pathway terms. The significance of fold change was evaluated with Benjamini-Hochberg adjusted p value < 0.01. The high-throughput sequencing data reported in this study have been deposited in Genome Sequence Archive (GSA), CRA001143.

### Chromatin immunoprecipitation (ChIP)

ChIP assay was performed using a magna ChIP™ kit (Merck, No: 17-10460) following the manufacture's protocol. Briefly, cell cultures (3 × 10^6^ cells) from mouse cortex (E20) were chemically cross-linked in the buffer A with a 1% final concentration of formaldehyde (No: ab185917, Abcam) in the presence of protease inhibitor cocktail II (No: ab201116, Abcam,). cells (5-10 × 10^6^ cells per experiment) were cross-linked in 1% formaldehyde (final concentration) at 20°C for 10 min. To quench cross-linking, glycine (100 µl/ml, No: ab120050, Abcam) was added and cells were washed three times with ice-cold PBS. Cells were then centrifuged for 5 min at 500 g, and re-suspended in ice cold buffer C. After 10 min incubation, pellet samples were centrifuged and re-suspended in 100 μl of the buffer D/PI mix. Shear DNA was generated by a sonicator to an optimal DNA fragment size of 200-1,000 bp and incubated with 1× ChIP elution buffer/PI mix and 5 μg anti-Hmgb2 antibody (No: ab124670, Abcam) or non-specific IgG and protein G magnetic beads overnight with rotation at 4°C. Beads were washed five times with RIPA buffer and once with TE buffer containing 50 mM NaCl. Bound complexes were eluted from the beads by heating at 65°C with occasional vortex and crosslinking was reversed by overnight incubation at 65°C. The chromatin fragments were purified and recovered by the Wizard® SV Gel and PCR Clean-Up System (A9281, Promega) and then qualification PCR was performed by primers of Hmgb2-binding sequences.

**Figure 3.**
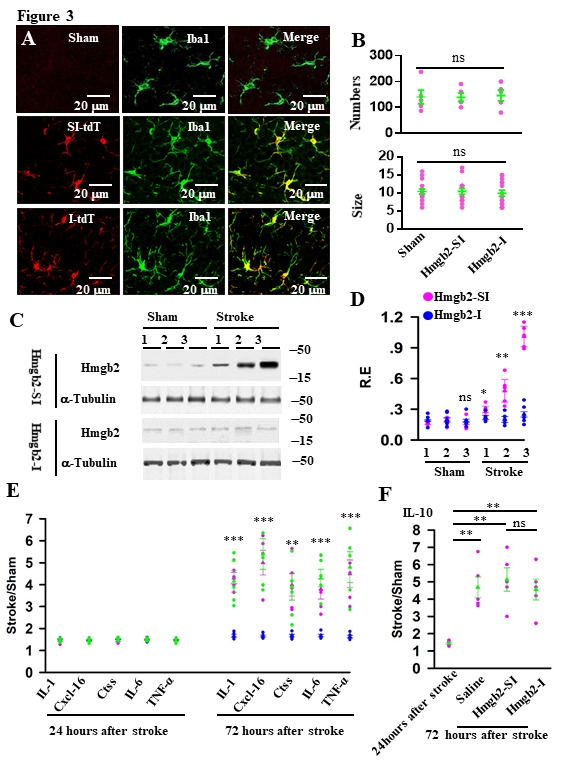
Hmgb2 mediates microglia pro-inflammatory response in stroke. (A) representative images show the expression of Hmgb2-SI (red, SI-tdT) and Hmgb2-I (red, I-tdT) in the cortex at 18 days after the injection of the AAV-PHP.eB-Hmgb2-SI/tdT or the AAV-PHP.eB-DIO-Hmgb2-I/tdT virus particles into the tail vein of the Cx3cr1-Cre mice. The sections are stained with anti-Iba1 (green). (B) the numbers and the soma size of the Iba1-labeted cells in the indivudal mice (circles) and their averages per group (triangles)at 18 days after the injection of AAV are plotted (ns = no significantly differences). (C) Hmgb2-I inhibits the Hmgb2 expression in microglia. The microglia lysates are prepared from mice expressing Hmgb2-SI or Hmgb2-I at 1, 2, or 3 days after operation with sham or stroke and blotted with anti-Hmgb2 or anti-α-tubulin, as indicated. (D) Relative expression (R.E) levels (defined by normalizing the band intensities of anti-Hmgb2 blots to the respective α-tubulin) in the individual mice (circles) and their averages per group (triangles) are plotted. Data are mean ± SEM (n = 5 mice per group, ns = no significantly differences, *p = 0.040; **p = 0.003; ***p < 0.0001 between Hmgb2-SI and Hmgb2-I, *t*-tests). (E) AAV-PHP.eB -DIO-Hmgb2-SI/tdT virus (green symbols) or AAV-PHP.eB -DIO-Hmgb2-I/tdT (blue symbols) virus particles or saline (pink symbols) were injected into the tail vein of the Cx3cr1-Cre mice. 18 days after the injection, mice were operated with sham or stroke. 24 or 72 hours after the operation, the ratios of IL-1, Cscl-16, Ctss, IL-6 and TNF-a in the extracellular space of stroke mice versus sham mice (stroke/Sham) are analyzed and plotted by the individual mice (circles) and their averages per group (triangles). Data are mean ± SEM (n = 5 mice per group, **p = 0.0041, ***p < 0.0001 between Hmgb2-SI and Hmgb2-I, *t*-tests). (F) the ratios of IL-10 in the extracellular space of stroke mice versus sham mice (stroke/Sham) are analyzed and plotted by the individual mice (circles) and their averages per group (triangles). Data are mean ± SEM (n = 5 mice per group, *F*_(3,16)_ = 9.506, ns = no significantly differences, **p = 0.0038, 0.0011, 0.0054, BF ANOVA).

### Luciferase reporters

The mouse *Ctss* promoter (1425 bp) was amplified by PCR from mouse genomic DNA and cloned into the firefly luciferase reporter plasmid (*pGL3-basic*, Promega), and co-transfected the renilla luciferase reporter plasmid (*p*RL-TK, Promega) as internal reference into HEK293 cells (3 × 10^5^ cells per well) with cDNA encoding *Hmgb2* (pcDNA3.1FlagmHmgb2, Addgene, plasmid: 31610) or enhanced green fluorescence protein (GFP). The empty vector (*p*cDNA3.2/V5, 500 ng) was used as a control. Cells were harvested and assayed for firefly and renilla luciferase activities using the dual-luciferase reporter assay system (E1910, Promega) according to the manufacturer's protocol. The data were normalized to the co-transfected β-galactosidase plasmid (Invitrogen) and expressed as the relative luciferase activity (units).

### Virus injection and cell sorting

The AAV-PHP.eB-DIO virus containing the encoding of Hmgb1-I(5’-GAGGCCTCCTTCGGCCTTC-3’), Hmgb1 -SI (5’-GTTGGTTCTAGCGCAGTTT-3’), Hmgb2-I (5’-GCCAAAGATAAACAACCGTAT-3’) or Hmgb2-SI (5’- GTCCAATAAGCTCTTTACAGT-3’) were created by Shanghai Taitool Bioscience Co.Ltd. 80 μl virus particles were injected into the tail vein of the Cx3cr1-Cre mice. 18 days after the injection, brain tissues were isolated, sliced and digested in buffer containing 10 mm Tris-Cl, pH 7.6, 50 mm NaF, 1 mm Na_3_VO_4_, 1 mm edetic acid, 1 mm benzamidine, 1 mm PMSF, 1 mg/10 ml papain, and a mixture of aprotinin, leupeptin, and pepstatin A (10 μg/ml each) for 30 min. Microglia expressed with GFP in the cell suspension was automatically isolated using an S3e Cell Sorter (Bio-Rad).

### RT-PCR analysis

Total RNA in the isolated microglia was extracted with TRIzol Reagent (15596026, Invitrogen) according to the manufacturer’s instructions. 1μg of the total RNA per sample was reverse transcribed using TaqMan® reverse transcription reagent (4366596, Invitrogen) to obtain cDNA products and amplified using the primers with the following sequences: *Hmgb2;* forward, AAAA GACCCCAATGCTCCGA, reverse, TCACCCAGTTTC TTCGCAGT, C*tss:* forward, ATACCAGGGTTCTTG TGGTGC; reverse, TGAGCAGTCCACCAGGTTCT; Hmgb1; forward, AGGCTGACAAGGCTCGTT, reverse, GATTTTGGGGCGGTACTCAG.

Hmgb2-binding sites of Ctss promoter: P1; forward, TTCTGTCCCTAGCAGCATGAC, reverse, CTGGAAC TGCCAACTTGTTTCA, P2; forward, CTGGCCAGAT GTTCTATTTCAGC, reverse, CTGGTGGTGCCGT ACACTC, P3; forward, AAACAAAGGAACT GATGA GTGAGT, reverse, TGATTCCCTAGGAAG CCCTGT.

### Western blot and ELISA analysis

To measure the secreted inflammatory factors in extracellular space, the brain tissue from mice were isolated and homogenized in ice-cold the buffer containing (250 mM sucrose, 20 mM HEPES, 10 mM KCl, 1 mM EDTA, 1 mM EGTA, 1 mM DTT and proteinase inhibitor mixture (Sigma, 5 μl/100 mg tissue) at pH 7.4. After centrifugation at 3, 000 r.p.m. for 5 min., the supernatants were separated and were used for the analysis of the secreted inflammatory factors in the extracellular space. Ctss and TNF-α proteins were detected using Western blots with anti-Ctss (1: 2000, HPA002988, Sigma) and anti-TNF-α (1: 3000, ab8348, Abcam), respectively. The levels of IL-1, Cxcl-16, IL-6, and TNF-α in the extracellular space were analyzed using enzyme linked immunosorbent assay (ELISA) with the following assay kits; mouse IL-1 beta ELISA kit (ab100704, Abcam), mouse Cxcl-16 ELISA kit (ab100677, Abcam), mouse IL-6 ELISA kit (ab100712, Abcam).

To analyze the expression levels of proteins and mRNA in microglia, the isolated microglia from the brain of mice, as described above were homogenized and diluted with a buffer containing 200 mm Tris-Cl, pH 7.6, 8% SDS, and 40% glycerol. The protein concentration was determined using a BCA kit (Pierce). Final concentrations of 10% β-mercaptoethanol and 0.05% bromophenol blue were added and the samples were boiled for 10 min in a water bath. The proteins in the extracts were separated by 10% SDS-PAGE and transferred to nitrocellulose membranes and blocked with 2% BSA in TBST for 1 h and then incubated overnight at 4 ºC with the specific primary antibodies, as the followings; rabbit monoclonal anti-Hmgb2 (1:1000, ab124670, Abcam), rabbit monoclonal anti-Hmgb1 (1:1000, ab79823, Abcam), rabbit anti-Ctss (1: 2000, HPA002988, Sigma), and mouse monoclonal anti-alpha-tubullin (1: 5000, No: 236-10501, Invitrogen). The membrane was then washed with TBST and incubated with the appropriate secondary antibodies (1:1000, Odyssey) for 1 h at room temperature (22 ± 1 ºC). After washing, the blots were scanned using an Infrared Imaging System (Odyssey, LI-COR). The band densities were quantitatively analyzed by using Kodak Digital Science 1-D software (Eastman Kodak).

### Immunohistochemistry

Mice were killed by intraperitoneally injection of an overdose of chloral hydrate and transcardially perfused with 100?mL saline (0.9% w/v NaCl) followed by 4% paraformaldehyde (PFA). Brains were removed and postfixed in 4% PFA. We cut 30-μm sagittal or horizontal sections (Leica Microsystems, Wetzlar, Germany). Immunohistochemistry was performed on free-floating brain sections as described previously^28, 34,35^. In brief, 30-μm free-floating coronal sections were stained and blocked in 3% normal goat serum at room temperature (20-22 °C) for 1?h. The sections were then incubated with one of the following primary antibodies: Iba1 (goat polyclonal anti-Iba1, 1: 1000, ab178846, Abcam), Hmgb2 (rabbit monoclonal anti-Hmgb2, 1:100, ab124670, Abcam), CD16/32 (Rat monoclonal antibody 1:300, 553141, BD bioscience) and reacted with conjugate-adsorbed Alexa Fluor 488 donkey anti-goat or Alexa Fluor 546 donkey anti-rabbit or Alexa Fluor 647 donkey anti-rat secondary antibodies. Sections were rinsed, dried, and cover-slipped with fluorescence mounting medium. Control sections were processed with omission of the primary antisera. Single-, double-, or triple-labeling was viewed and imaged with a confocal laser-scanning microscope (Zeiss LSM800 Examiner Z1) and analyzed with a three-dimensional constructor (Image-Pro Plus software). A series of confocal images was taken at 0.5-μm intervals through the region of interest, and optical stacks of 6-12 images were produced for the figures. For cell counting, experimenters coded all slides from the experiments before quantitative analysis. Quantification was performed by the other experimenters who were unaware of the experimental conditions and treatments.

**Figure 4.**
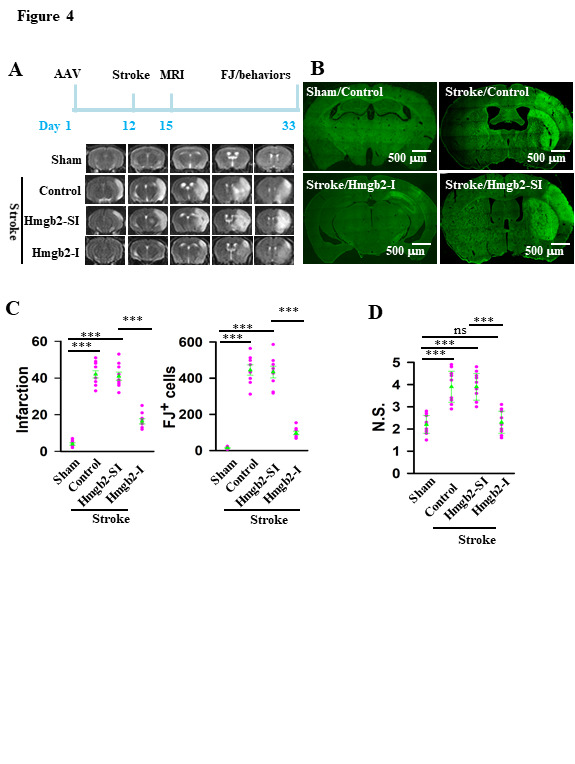
Inhibition of Hmgb2 protects against stroke damages. (A) the experimental schedules (top) and representative images (bottom) show MRI imaging from sham and stroke mice without (control) or with the expression of Hmgb2-SI or Hmgb2-I. (B) the representative images show FJ labeling from sham and stroke mice without (control) or with the expression of Hmgb2-SI or Hmgb2-I. (C) the infarction size (mean ± SEM, n = 9 mice per group, *F*_(3, 32)_ = 129, ns = no significantly differences, ***p < 0.0001; BF ANOVA) and the number of FJ^+^ cells (mean ± SEM, n = 9 mice per group, *F*_(3, 32)_ = 134.5, ns = no significantly differences, ***p < 0.0001; BF ANOVA) in the individual mice (circles) and the averages per group (triangles) are plotted. (D) inhibition of Hmgb2 improves the neurological functions of stroke mice. The neurological scores (N.S) of the individual mice (circles) and the averages per group (triangles) are plotted. Data are mean ± SEM (n = 9 mice per group, *F*_(3, 32)_ = 23.9, ns = no significantly differences, ***p < 0.0001; BF ANOVA).

### LHVS injection and cysteine protease assay

LHVS was synthesized by Peptide Institute, INC (Osaka, Japan) and solubilized in 20% cremophor EL (61791-12-6, Sigma) and injected (2 μl per injection at a concentration of 5 mg/ml per day for 6 consecutive days) directly into the third ventricle of mice on the second days after operation with sham or stroke. After the final injection, brain tissues were isolated from mice and homogenized ice-cold buffer from a Ctss assay kit (ab65307, Abcam). Cysteine protease activity was measured according to the manufacturer’s instructions. Mice without (control) or with the injection of vehicle (20% cermophor EL) were used as the controls. The ratios of protease activity in stroke mice versus the respective sham mice were calculated.

### Measurements of blood brain barrier function

To analyze blood brain barrier permeability, mice were injected with 4% Evans blue dye (EB, 314-13-6, Sigma-Aldrich, St. Louis, MO, USA) at 4 ml/kg of body weight through the lateral tail vein 7d after operation with sham or stroke. One hour after the injection, mice were transcranially perfused with 100 ml PBS and the brains were removed. Contralateral hemisphere to the occlusion were separated and weighed. Hemisphere samples were homogenized in 50% trichloroacetic acid (T0699, Sigma-Aldrich, St. Louis, MO, USA) solution and centrifuged (15 min, 13000g, 4°C). The supernatant was diluted with three times volume of absolute ethanol and measured by spectrofluorometer (Thermo Scientific, Waltham, MA, USA) at 620 nm. The amount of EB in the brain tissue (ng/g tissue) was quantified.

**Figure 5.**
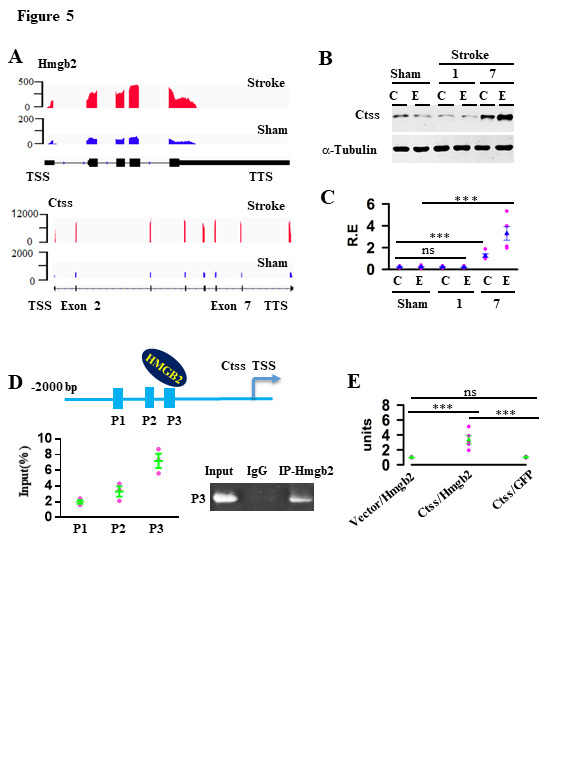
HMGB2 promotes the transcription of Ctss. (A) patterns of Hmgb2 and Ctss transcripts in the cortex of mice at 3 days after operation with stroke, as compared to sham. (B) the lystaes of cytosolic fraction and extracellular space from mice at 1 or 7 days after operation with sham or stroke were blotted with anti-Ctss or α-tubulin, as indicated. C, Relative expression (R.E) levels (defined by normalizing the band intensities of anti-Ctss blots to the respective α-tubulin) in the individuals (circles) and their averages per group (triangles) are plotted. Data are mean ± SEM (n = 5 mice per group, ns = no significantly differences, ***p < 0.0001, *t*-tests). (D) illustration (top) shows a direct binding of Hmgb2 to a promoter region of Ctss. CHIP- qualificative PCR assay shows amplification of the predicted Hmgb2-binding sites in Ctss promoter precipitated with anti-Hmgb2 or non-specific IgG, Relative expresssion of each site in the individuals (circles) and their averages per group (triangles) are plotted (left), n=3, representative image shows the bands of P3 PCR product (right) . (E) The relative luciferase activity (units) in the indiviudal assay (circles), in which a full length (1425 bp)of Hmgb2 binding sites located in Ctss promoter or GFP was co-expressed with an empty vector or Hmgb2, and their averages per group (triangles) are plotted (bottom). Data are mean ± SEM (n = 5 assays per group, *F*_(2, 12)_ = 18.72, ns = no significantly differences, ***p < 0.0001, BF ANOVA).

### Statistical analysis

All values in the text and figure legends are presented as mean?±?s.e.m. Unpaired two-tailed Student’s *t* tests (*t* test) were used in Western blots, and repeated one-way ANOVA and post hoc Bonferroni corrections following two-way analyses of variance (BF ANOVA) were used in all behavioral tests were used when assumptions of normality and equal variance (*F* test) were met. Data distribution was assumed, but this was not formally tested. Significance set at *P*?<?0.01. All behavioral tests were performed by experimenters blinded to the experimental conditions. Analysis of RNA-sequencing DEGs was calculated by negative binomial test with FDF correction. The significance of fold change enrichment was calculated using Benjamini-Hochberg adjusted *p* < 0.01. For all other experiments, data collection and analysis were not performed blind to the conditions of the experiments. Power calculations were performed using G*power software v3.1.9.2 (IDRE Research Technology Group, Los Angeles, USA). Group sizes were estimated based on recent studies and were designed to provide at least 80% power with the following parameters: probability of type-I error (α)?=?0.05, a conservative effect size of 0.25, and 3-8 treatment groups with multiple measurements obtained per replicate.

## RESULTS

### Genome-wide expression differences across the time course after stroke

To identify the microglia pro-inflammatory mediators in stroke, we operated adult male mice at 120 ± 5 days old of age with sham or middle cerebral artery occlusion, which is widely used as an experimental model of stroke[7]. Brain damages were analyzed using magnetic resonance image (MRI, Fig. 1A) and 2, 3, 5-triphenyltetrazolium chloride (TTC, Fig. 1B) and Fluoro-Jade (FJ, Fig. 1C, D) staining. We next assessed the gene expression patterns during the course of stroke damages using the next-generation of RNA sequencing. We isolated the total RNAs from the cortex of mice at 1, 3, 7, 14 or 28 days after operation with sham or stroke. RNA sequencing libraries were established and profiled by illumination high-throughput RNA sequencing (Fig. 1E, Supplementary Table 1). We obtained more than 100 million paired-ed Clean reads. The Q30 value of each sample Clean reads is greater than 88% and the total mapping rate of each sample is greater than 80%, which indicates that accuracy and repeatability of sequencing data were fitted for the next analysis (Supplementary Table 1-2). All data presented are freely available without restriction in the public open access database (Genome Sequence Archive (GSA), CRA001143).

We subsequently analyzed differentially expressed genes (DEGs) between sham and stroke mice with a cutoff of at least twofold and *p* value < 0.05. We found that stroke increased the expression of a total of 778 protein coding genes (Supplementary Tables 3-5). We classified these DEGs into three different clusters in terms of the expression differences across the time course after stroke onset and their biological functions through gene ontology (GO) analysis (Fig. 1F-G) Cluster one genes were expressed at the first day after stroke onset (Fig. 1E, Supplementary Table 3). A large fraction of these genes such as Hmgb2, TNF and IL-11 were pro-inflammatory factors Cluster two genes such as Ctss and PTPN18are either peptidases or proteases that were expressed at 3 days and peaked 7 days after stroke onset (Fig. 1E, Supplementary Table 4). A total of 190 genes such as complement component 3 (C3) and C6 were expressed in the brain cells 14 days after stroke onset and peaked 14 days later (Fig. 1E, Supplementary Table 5). These genes were designated as cluster three genes, which are largely located in the GO categories that modulate both innate and adaptive immune responses [9]. Here, we focus on the major microglia inflammatory response mediators in stroke of cluster one and cluster two genes.

### The expression of Hmgb2 and pro-inflammatory factors in microglia are increased during stroke

We next validated the roles of Hmgb2 in stroke damages.Hmgb2 is a member of high mobility group protein family (Hmgb1-Hmgb4) proteins that influence microglia pro-inflammatory response in some neurological disorders [10]. But, whether Hmgb2 plays roles in stroke damages is yet to be studied. To explore the expression of Hmgb2 in the microglia after stroke, we co-stained Hmgb2 with Iba1 by immunofluorescence at 3days after stroke. Significantly, Hmgb2 highly expressed in the activated microglia after stroke compared with the sham (Fig. 2A). Then we assessed the Hmgb2 level changed in microglia separately. We expressed ZsGreen1, an enhanced green fluorescent protein variant, selectively in microglia by cross the Cx3cr1-cre mice with Ai6 mice (Jax. Stock No:007906), which express robust Zsgreen1 fluorescence following cre mediated recombination. Fluorescence-activated cell sorting (FACS) was used to separate the microglia from cerebral cortex of the brain at 0, 12, 24, 48, 72 hours after stroke respectively. Western blots of the lysates from microglia showed that the levels of Hmgb2 in stroke mice were elevated by more than 6-fold of sham controls (0.17 ± 0.024 in sham versus 1.02 ± 0.13 in stroke group, mean ± SEM, n = 5 mice/per group, *p* = 0.0021, *t*-tests, Fig. 2B, C).

**Figure 6.**
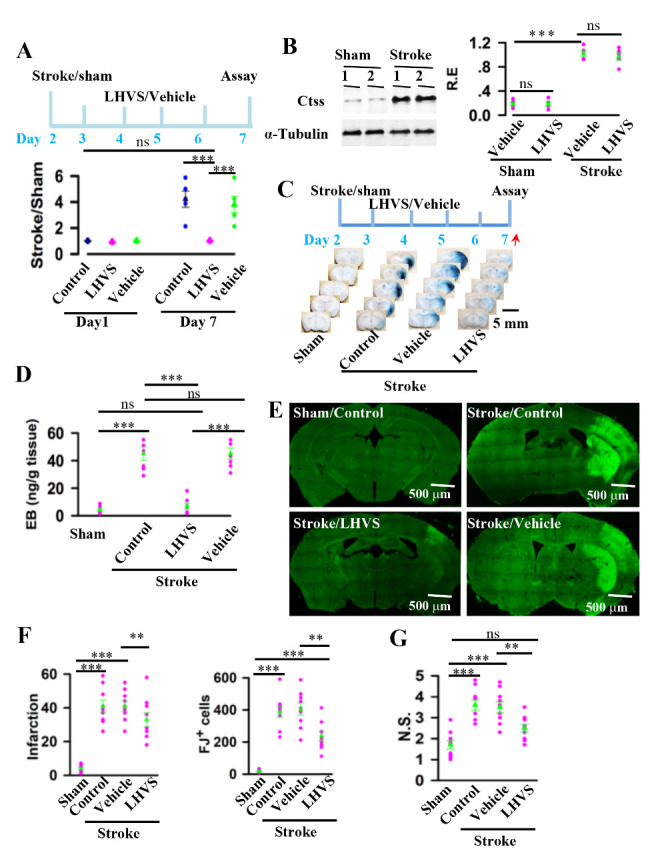
Inhibition of Ctss protects against stroke damages. (A) illustration (top) shows the experimental schedules of the consecutive injections of LHVS or vehicle directly into the third ventricle of mice on the second day after operation with sham or stroke. Ctss protease activity (defined by the ratios between stroke versus sham mice) in the individuals (circles) and their averages per group (triangles) ares plotted (bottom). Data are mean ± SEM (n = 5 mice per group, *F*_(2, 12)_ = 11.35, ns = no significantly differences, ***p < 0.0001, BF ANOVA). (B) mice were operated with sham or stroke. On the second day after operation, mice were injected with LHVS or vehicle once per day for 6 consecutive days. After the last injection, the cell lysates from the cortex were preapred and blotted with anti-Ctss or α-tubulin, as indicated. Relative expression (R.E) levels (defined by normalizing the band intensities of anti-Ctss blots to the respective α-tubulin) in the individuals (circles) and their averages per group (triangles) are plotted. Data are mean ± SEM (n = 5 mice per group, ns = no significantly differences, ***p < 0.0001, *t*-tests). (C) illustration (top) shows experimental schedules of EB injection, as indicated by red arrow, at 7 days after operation with sham or stroke. representative images (down) show that the presence of Evans blue dye (EB) in the brain of stroke mice after operation with sham or stroke. (D) The amounts of EB in the brain tissue of the individual mice (circles) and their averages per group (triangles) are plotted. Data are mean ± SEM (n = 5 mice per group, *F*_(3, 24)_ = 44.37, ns = no significantly differences, ***p < 0.0001, BF ANOVA). (E) representative FJ labeling from sham and stroke mice without (control) or with the application of LHVS or vehicle. (F) the infarction (mean ± SEM, n = 9 mice per group, *F*_(3, 32)_ = 31.9, ns = no significantly differences, ***p < 0.0001, BF ANOVA) and the FJ^+^ cells (mean ± SEM, n = 9 mice per group, *F*_(3, 32)_ = 35.93, ns = no significantly differences, ** *p* =0.0025, ***p < 0.0001; BF ANOVA) in the individual mice (circles) and the averages per group (triangles) are plotted. G, inhibition of the protease activity of Ctss improves the neurological functions of stroke mice. The neurological scores (N.S) of the individual mice (circles) and the averages per group (triangles) are plotted. Data are mean ± SEM (n = 9 mice per group, *F*_(3, 32)_ = 16.06, ns = no significantly differences, ** *p* = 0.0069, ***p < 0.0001, BF ANOVA).

Four isoforms of Hmgb1-Hmgb4 with a shared structural motif have been reported[10]. Among them, Hmgb1 and Hmgb2 share 80% sequence homology. We found, however, that neither mRNA nor protein of Hmgb1 was changed in microglia of stroke mice, as compared to the controls (Fig. S1A-C), indicating that stroke selectively induces the expression of Hmgb2. We noticed that Hmgb2 elevation was closely associated with an increase in both the numbers (125 ± 13 versus 258 ± 27, mean ± SEM, n = 5, *p* = 0.013, *t*-test, Fig. 2D, E) and the size of microglia (10 ± 0.7 versus 23 ± 2, mean ± SEM, n = 15 cells/5 mice per group, *p* <0.0001, *t*-test, Fig. 2D, E), and the production of pro-inflammatory factors such as IL-1, C-X-C motif chemokine ligand (Cxcl)-16 and TNF-α (n = 5 mice per group, **p = 0.0091, 0.014, 0.0059, ***p < 0.0001, *t*-tests, Fig.2F). These results indicated that Hmgb2 might play a role in microglia pro-inflammatory response after stroke.

### Inhibition of Hmgb2 protects against microglia pro-inflammatory response

We next determined whether inhibition of Hmgb2 protects against microglia pro-inflammatory response. We expressed a small interference RNA that specifically targets Hmgb2 gene (Hmgb2-I) in microglia of adult mice. We generated type-PHP.eB recombinant adeno-associated virus (AAV-PHP.eB-DIO-Hmgb2-I/tdT virus) particles which could cross the blood-brain barrier. A scrambled small interference RNA (Hmgb2-SI) was used as a control. A high titer (80 μl, 5 × 10^11^ genomic particles/ml) of the virus particles were injected into the tail vein of Cx3cr1-Cre mice, immunofluorescence of Iba1 shows that the virus expressed in microglia specifically (Fig. 3A) without affecting the activation of microglia (Fig. 3B). We also injected the virus into Cx3cr1-Cre*Ai6 mice, 18 days after the virus injection, mice were operated with sham or stroke. Microglia were separated from cerebral cortex of the brain after stroke by FACS and lysates from microglia were used to detect the relative expression of Hmgb2 by western blots. Our data showed that application of Hmgb2-I, but not Hmgb2-SI, knocked down the Hmgb2 expression in microglia after stroke onset as expected (Fig. 3C, D). In addition, Hmgb2-I also inhibited the number of M1 microglia by co-stained Iba1 and CD16/32, an M1 microglia marker (Fig. S2), and the pro-inflammatory response in stroke mice, as determined by a complete inhibition of the production of IL-1, Ctss, TNF-α, Cxcl-16 and IL-6 (Fig. 3E), but not the production of anti-inflammatory signals such as IL-10 in stroke mice (Fig. 3F).

### Inhibition of Hmgb2 specifically protects against stroke damage

Considering the pro-inflammatory response in microglia was suppressed by the Hmgb2-I virus, we next checked whether the brain damages caused by stroke can be ameliorated. We observed that the loss of function resulting from application of Hmgb2-I effectively ameliorated brain damages of stroke mice in both the MRI imaging (16.44 ± 1.45 versus 41.22± 2.22, mean ± SEM, *F*_(3, 32)_ = 129, n = 9, *p* <0.0001, BF-ANOVA, Fig 4A, C), FJ^+^ cell number (91 ± 6.87 versus 433.33 ± 28.30, mean ± SEM, *F*_(3, 32)_ = 134.5, n = 9, *p* <0.0001, BF-ANOVA, Fig 4B, C) and significantly improved the neurological functions (2.3 ± 0.2 versus 4.0 ± 0.2, mean ± SEM, *F*_(3, 32)_ = 23.9, n = 9, *p* <0.0001, BF-ANOVA, Fig. 4D). Silencing Hmgb1 gene using a small interference RNA that specifically targets Hmgb1 gene (Hmgb1-I, Fig. S3A-C), but not Hmgb2 (Fig. S4) produced no protective effects on stroke damages (Fig. S5). Thus, Hmgb2, but not Hmgb1, functions as a microglia activator and mediates microglia pro-inflammatory response in stroke and inhibition of Hmgb2 in microglia specifically protects against pro-inflammatory response and stroke damage

### HMGB2 promotes the transcription of ctss

Among these genes, Ctss was interested as it encodes a cathepsin S cysteine protease[11]. Ctss was mainly expressed in microglia[12] and the transcriptional level of Ctss after stroke was correlated with the expression of Hmgb2 (Fig. 2F, 5A). Besides, inhibition of Hmgb2 in microglia decreased the content of Ctss at 3 days after stroke operation (Fig. 3E). To confirm the relationship between Hmgb2 and Ctss, we first verified the relative expression (R.E) of Ctss in cytosolic fraction (C) = 0.2 ± 0.03 in sham versus 1.26 ± 0.15 in 7 days after stroke (mean ± SEM, *F*_(2,12)_ = 36.51, ***p < 0.0001, BF ANOVA, Fig. 5B, C) and secreted into extracellular space after stroke onset; the relative expression (R.E) in extracellular space (E) = 0.23 ± 0.04 in sham versus 3.3 ± 0.62 in 7 days after stroke (mean ± SEM, *F*_(2, 12)_ = 24.54, ***p = 0.0002, BF ANOVA, Fig. 5B, C). We next predicted whether Hmgb2 can regulate Ctss expression by JASPAR (http://jaspar.genereg.net), an open-access database of transcription factor binding profiles[13]. As shown in Fig. 5D, Hmgb2 can bind the promoter sequence of Ctss through several high score Hmgb2-bing sites (P1, P2, P3). Then, we validated that Hmgb2 was preferred to bind to the P3 site of Ctss promoter through ChIP-qualification PCR (Fig. 5D). We next cloned the promoter sequence of Ctss which included the all Hmgb2 binding sites into luciferase reporter vectors. Data shows Hmgb2 overexpression enhanced the luciferase activity. The relative luciferase activity (R.L.A) = 0.99 ± 0.03 in empty vector versus 3.36 ± 0.54 in Ctss promoter (mean ± SEM, *F*_(2,12)_ = 18,72, ns = no significantly differences, ***p < 0.0001, BF ANOVA, Fig. 5E). Thus, we verified that Ctss was a pro-inflammatory mediator downstream of Hmgb2.

### Inhibition of protease activity of Ctss restores the blood-brain barrier function and stroke damage

Secretion of Ctss was associated with blood brain barrier dysfunction in stroke mice. To determine functional implications of Ctss expression and secretion in the stroke-induced blood brain barrier dysfunction, we used morpholinurea-leucine-homophenylalanine-vinyl phenyl sulfone (LHVS), which is an irreversible Ctss inhibitor[14]. LHVS was injected (2 μl per injection at a concentration of 5 mg/ml per day for 6 consecutive injections) directly into the third ventricle of mice on the second days after operation with sham or stroke. After the final application, protease activity was analyzed. Mice without or with the injection of vehicle were used as the controls. LHVS was effective at inhibition of protease activity of Ctss (4.2 ± 0.6 in control versus 1.03 ± 0.03 in LHVS; mean ± SEM, *F*_(2, 12)_ = 11.35, ns = no significantly differences, ***p < 0.0001, BF ANOVA, Fig. 6A). Application of LHVS did not influence the levels of Ctss protein (Fig. 6B), but it restored the blood-brain barrier function in stroke mice (6.93 ± 2.24 versus 45.29 ± 4.14, mean ± SEM, *F*_(3, 24)_ = 44.37, n = 9, *p* <0.0001, BF-ANOVA, Fig. 6C, D) and protected the brain cells against the stroke injuries (FJ^+^ cell number: 238.22 ± 30.21 versus 405.11 ± 38.41, mean ± SEM, *F*_(3, 32)_ = 35.93, n = 9, *p* =0.0025, BF-ANOVA; Infarction area: 19.56 ± 2.31 versus 34.78 ± 3.21, mean ± SEM, *F*_(3, 32)_ = 31.9, n = 9, *p* =0.0014, BF-ANOVA, Fig 6E, F) and significantly improved the neurological functions (2.5 ± 0.2 versus 3.5 ± 0.3, mean ± SEM, *F*_(3, 32)_ = 16.06, n = 9, *p* =0.0069, BF-ANOVA, Fig. 6G). Thus, Ctss is a pro-inflammatory signal downstream of Hmgb2 and contributes to the blood brain barrier dysfunctions in stroke.

**Figure 7.**
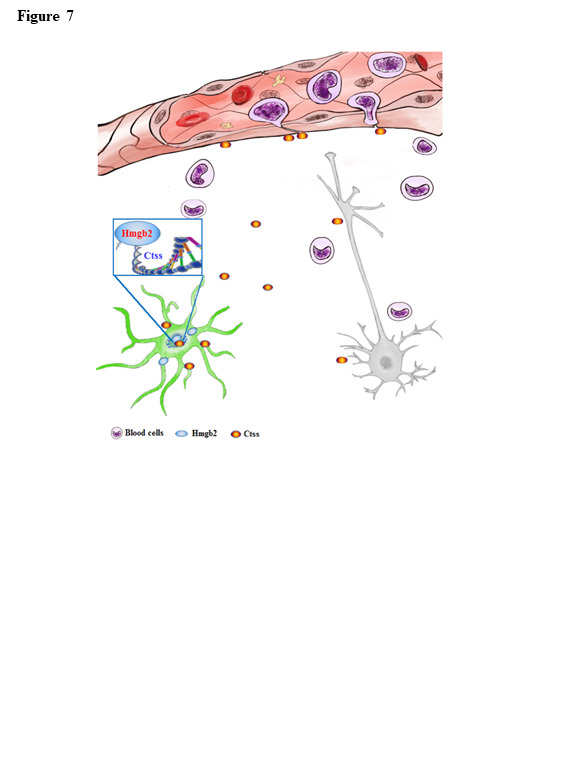
Working model: stroke activates microglia inflammatory response via Hmgb2. Stroke induces the expression of Hmgb2 in microglia and in turn causes microglia proliferation and morphoslogical changes. Hmgb2 in the nucleus of microglia binds to a promoter region of Ctss and activates Ctss transcription, resulting in the expression and secretion of Ctss in the extracellular space. Ctss is a cycteine protease and breaks blood brain barrier, leading to the entrance of blood cells into the brain, which causes the secondary brain infarction.

## DISCUSSION

This study has reported a discovery that Hmgb2 acts as a microglia activator and mediates microglia pro-inflammatory and immune response in stroke. Stroke activates microglia in the brain and produces both pro- and anti-inflammatory responses. Pro-inflammatory response causes brain cell death[3], whereas anti-inflammatory events involve the cellular survival and the tissue repairing[4]. The studies about the dynamic transition of microglia from anti-inflammatory phenotype (M2) to pro-inflammatory events suggested that it is essential to balance the dual and opposing roles of microglia[5].

In our present study, using the genome-wide screening strategies we have identified a total of 778 genes that are expressed in the brain throughout the course of stroke damages. Of these, Hmgb2 belongs to the high mobility group box (HMGB) family which is one of danger-associated molecular patterns (DAMPs) released by necrotic cells. With the abundant non-histone DNA binding site, Hmgb1 can combine with DNA and intracellular proteins to mediate DNA repair and transcription[15]. Various evidences demonstrate that Hmgb1 is released after cytokine stimulation and function as a pro-inflammatory cytokine in stroke[16, 17]. But the function and underlying mechanisms of Hmgb2 in stroke remain unclear. We demonstrated that Hmgb2 is primarily expressed in microglia and mediates microglia pro-inflammatory response after stroke. Thus, Hmgb2 is a promising target for therapeutic intervention of stroke as inhibition of Hmgb2 selectively blocks microglia pro-inflammatory signals blocked while preserving microglia anti-inflammatory functions.

Following microglia activation, blood brain barrier becomes damages, resulting in the entrance of harmful substances from peripheral system into the brain[18]. In the present study we have shown that Hmgb2 activates Ctss transcription and induces Ctss expression in microglia of stroke mice. Ctss is a lysosomal protease and a member of the cysteine cathepsin family which are associated a range of pathological conditions and substrates such as major histocompatability complex class II(MHCII) and junctional adhesion molecule-B (JAM-B) involved blood brain barrier metastases[19]. Cathepsin S can be controlled in transcriptional level since the Ctss gene contains several transcription regulatory sites including Sp1, Sp3 and interferon-stimulated response element(IRSE)[20, 21]. Beside, restricted tissue expression in macrophages and a broad pH profile of Cathepsin S indicate it could be an ideal target for disease[22].After stroke onset, Ctss is secreted from microglia onto the extracellular space and subsequently breaks down the blood brain barrier. Inhibition of Ctss restores the blood brain barrier function and produces the therapeutic effects against stroke damages. Thus, Ctss as a microglia pro-inflammatory mediator downstream of Hmgb2 contributes to the blood brain barrier dysfunction of stroke. Yet, little is known about the mechanisms for how microglia pro-inflammatory response causes stroke damages. Our present findings reveal a plausible explanation that stroke induces Hmgb2 expression in microglia and initiates microglia pro-inflammatory response including the expression and secretion of Ctss onto the extracellular space in the brain. Ctss secretion causes subsequently blood brain barrier dysfunction, resulting in the cell death and the brain damages (Fig. 7). Thus, inhibition of either Hmgb2 or Ctss is effective for therapeutic intervention of stroke.

## Supplementary Materials

The Supplemenantry data can be found online at: www.aginganddisease.org/EN/10.14336/AD.2020.0926.


