# Supplementary material for: Genome-Wide Screen and Validation of Microglia Pro-Inflammatory Mediators in Stroke
Source: Aging Dis. 2021 Jun 1;12(3):786–800. doi: 10.14336/AD.2020.0926 (PMC8139211; doi:10.14336/AD.2020.0926)
Supplement: Supplementary file 1 [file AD-12-3-786-s.pdf]

SUPPLEMENTARY DATA

# Genome-Wide Screen and Validation of Microglia Pro-Inflammatory Mediators in Stroke

**Jianhua Wu[1,2,3,#], Zhuoze Wu[1,2,#], Aodi He[1,2], Tongmei Zhang[1,2], Ping Zhang[2,4], Jing Jin[1,2], Sisi Li[1,2], Gaigai Li[2,4], Xinyan Li[1,2], Shiqi Liang[1,2], Lei Pei[,2.5], Rong Liu[2.6], Qing Tian[2,6], Ximiao He[1], Youming Lu[1,2], Zhouping Tang[2,4*], Hao Li[1,2*]**

© 2020. Wu J et al. Published online at http://www.aginganddisease.org/EN/10.14336/AD.2020.09206

# SUPPLEMENTARY DATA

**Supplementary Table 1.** Quality control for RNA sequencing.

| Sample name | Clean reads | Clean bases | Error rate (%) | Q20 (%) | Q30 (%) | GC content (%) |
|---|---|---|---|---|---|---|
| Sham 1D-1 | 131417424 | 19.71G | 0.02 | 96.61 | 91.28 | 49.33 |
| Sham 1D-2 | 152761736 | 22.91G | 0.02 | 95.79 | 89.81 | 53.66 |
| Sham 1D-3 | 161623258 | 24.24G | 0.02 | 95.79 | 89.8 | 54.35 |
| Sham 3D-1 | 154387366 | 23.16G | 0.02 | 95.88 | 89.97 | 54.4 |
| Sham 3D-2 | 152080990 | 22.81G | 0.02 | 95.59 | 89.57 | 54.68 |
| Sham 3D-3 | 150211120 | 22.53G | 0.02 | 96.02 | 90.35 | 53.32 |
| Sham 7D-1 | 133329410 | 20G | 0.02 | 96.44 | 91.06 | 48.9 |
| Sham 7D-2 | 176385470 | 26.46G | 0.02 | 95.96 | 90.19 | 53.63 |
| Sham 7D-3 | 139356522 | 20.9G | 0.02 | 96.68 | 91.4 | 48.25 |
| Sham 14D -1 | 138656436 | 20.8G | 0.02 | 96.16 | 91.03 | 47.67 |
| Sham 14D -2 | 161505522 | 24.23G | 0.01 | 97.3 | 93.12 | 48.63 |
| Sham 14D-3 | 160132972 | 24.02G | 0.02 | 95.98 | 90.76 | 51.2 |
| Sham 28D -1 | 148152290 | 22.22G | 0.02 | 95.89 | 90.59 | 50.98 |
| Sham 28D -2 | 138897104 | 20.83G | 0.02 | 96.14 | 91.01 | 48.39 |
| Sham 28D -3 | 181555252 | 27.23G | 0.01 | 98.14 | 95.17 | 47.08 |
| Stroke 1D-1 | 151527848 | 22.73G | 0.02 | 95.77 | 89.86 | 55.9 |
| Stroke 1D-2 | 151070176 | 22.66G | 0.02 | 95.75 | 89.81 | 54.91 |
| Stroke 1D-3 | 165881914 | 24.88G | 0.02 | 97.11 | 92.62 | 48.62 |
| Stroke 3D-1 | 159735374 | 23.96G | 0.02 | 95.46 | 89.36 | 53.77 |
| Stroke 3D-2 | 147253844 | 22.09G | 0.02 | 96.04 | 90.28 | 52.46 |
| Stroke 3D-3 | 170407598 | 25.56G | 0.02 | 95.86 | 90 | 50.63 |
| Stroke 7D-1 | 178364232 | 26.75G | 0.02 | 96.57 | 91.2 | 50.75 |
| Stroke 7D-2 | 164376752 | 24.66G | 0.02 | 96.05 | 90.46 | 52.87 |
| Stroke 7D-3 | 185886506 | 27.88G | 0.02 | 95.19 | 88.83 | 55.45 |
| Stroke 14D -1 | 160705286 | 24.11G | 0.02 | 97.12 | 92.56 | 51.97 |
| Stroke 14D -2 | 140030994 | 21G | 0.02 | 97.2 | 92.83 | 51.3 |
| Stroke 14D -3 | 147363924 | 22.1G | 0.02 | 97.12 | 92.52 | 48.52 |
| Stroke 28D -1 | 163534926 | 24.53G | 0.01 | 97.36 | 93.22 | 48.64 |
| Stroke 28D -2 | 181231614 | 27.18G | 0.02 | 97.05 | 92.48 | 51.79 |
| Stroke 28D -3 | 135746966 | 20.36G | 0.02 | 97.13 | 92.65 | 51.6 |

Mice were operated with sham or stroke. Total RNAs from the cerebral cortex of mice were then prepared at 1, 3, 7, 14, or 28 days after the operation. Each operation at each time point has three replicates. Samples are then named as the followings: sham or stroke 1D-1, 1D-2, 1D-3, 3D-1, 3D-2, 3D-3, 7D-1, 7D-2, 7D-3, 14D-1, 14D-2, 14D-3, and 28D-1, 28D-2 and 28D-3. A total of 5 μg RNA from each sample was used as the input material for RNA sequencing.

© 2020. Wu J et al. Published online at http://www.aginganddisease.org/EN/10.14336/AD.2020.09206

# SUPPLEMENTARY DATA

**Supplementary Table 2.** RNA sequencing data mapping.

| Sample name | Total reads | Total mapped | Multiple mapped | Uniquely mapped |
|---|---|---|---|---|
| Sham 1D-1 | 131417424 | 127236362 (96.82%) | 12270319 (9.34%) | 114966043 (87.48%) |
| Sham 1D-2 | 152761736 | 130200862 (85.23%) | 9320603 (6.1%) | 120880259 (79.13%) |
| Sham 1D-3 | 161623258 | 138804703 (85.88%) | 11219765 (6.94%) | 127584938 (78.94%) |
| Sham 3D-1 | 154387366 | 133499964 (86.47%) | 9464303 (6.13%) | 124035661 (80.34%) |
| Sham 3D-2 | 152080990 | 126313080 (83.06%) | 9549787 (6.28%) | 116763293 (76.78%) |
| Sham 3D-3 | 150211120 | 129840391 (86.44%) | 7682654 (5.11%) | 122157737 (81.32%) |
| Sham 7D-1 | 133329410 | 123031384 (92.28%) | 4877298 (3.66%) | 118154086 (88.62%) |
| Sham 7D-2 | 176385470 | 153587791 (87.08%) | 9500613 (5.39%) | 144087178 (81.69%) |
| Sham 7D-3 | 139356522 | 134932112 (96.83%) | 10791067 (7.74%) | 124141045 (89.08%) |
| Sham 14D -1 | 138656436 | 131947602 (95.16%) | 13012087 (9.38%) | 118935515 (85.78%) |
| Sham 14D -2 | 161505522 | 150477259 (93.17%) | 9770144 (6.05%) | 140707115 (87.12%) |
| Sham 14 D-3 | 160132972 | 137970956 (86.16%) | 10050583 (6.28%) | 127920373 (79.88%) |
| Sham 28D -1 | 148152290 | 125305893 (84.58%) | 11503954 (7.76%) | 113801939 (76.81%) |
| Sham 28D -2 | 138897104 | 132348990 (95.29%) | 11657889 (8.39%) | 120691101 (86.89%) |
| Sham 28D -3 | 181555252 | 170518491 (93.92%) | 9618227 (5.3%) | 160900264 (88.62%) |
| Stroke 1D-1 | 151527848 | 126050834 (83.19%) | 9452057 (6.24%) | 116598777 (76.95%) |
| Stroke 1D-2 | 151070176 | 125899943 (83.34%) | 11320496 (7.49%) | 114579447 (75.85%) |
| Stroke 1D-3 | 165881914 | 153989628 (92.83%) | 11032950 (6.65%) | 142956678 (86.18%) |
| Stroke 3D-1 | 159735374 | 135676871 (84.94%) | 10835260 (6.78%) | 124841611 (78.16%) |
| Stroke 3D-2 | 147253844 | 129684629 (88.07%) | 6216317 (4.22%) | 123468312 (83.85%) |
| Stroke 3D-3 | 170407598 | 154375539 (90.59%) | 11482189 (6.74%) | 142893350 (83.85%) |
| Stroke 7D-1 | 178364232 | 159705985 (89.54%) | 8903276 (4.99%) | 150802709 (84.55%) |
| Stroke 7D-2 | 164376752 | 144339390 (87.81%) | 8020995 (4.88%) | 136318395 (82.93%) |
| Stroke 7D-3 | 185886506 | 154787176 (83.27%) | 9252976 (4.98%) | 145534200 (78.29%) |
| Stroke 14D -1 | 160705286 | 141552949 (88.08%) | 9502889 (5.91%) | 132050060 (82.17%) |
| Stroke 14D -2 | 140030994 | 135539772 (96.79%) | 23388171 (16.7%) | 112151601 (80.09%) |
| Stroke 14D -3 | 147363924 | 143045816 (97.07%) | 10506738 (7.13%) | 132539078 (89.94%) |
| Stroke 28D -1 | 163534926 | 151765206 (92.8%) | 11506037 (7.04%) | 140259169 (85.77%) |
| Stroke 28D -2 | 181231614 | 155068798 (85.56%) | 12488137 (6.89%) | 142580661 (78.67%) |
| Stroke 28D -3 | 135746966 | 120278993 (88.61%) | 8602710 (6.34%) | 111676283 (82.27%) |

Mice were operated with sham or stroke. Total RNAs from the cerebral cortex of mice were then prepared at 1, 3, 7, 14, or 28 days after the operation. Each operation at each time point has three replicates. Samples are then named as the followings: sham or stroke 1D-1, 1D-2, 1D-3, 3D-1, 3D-2, 3D-3, 7D-1, 7D-2, 7D-3, 14D-1, 14D-2, 14D-3, and 28D-1, 28D-2 and 28D-3. A total of 5 μg RNA from each sample was used as the input material for RNA sequencing.

© 2020. Wu J et al. Published online at http://www.aginganddisease.org/EN/10.14336/AD.2020.09206

# SUPPLEMENTARY DATA

**Supplementary Table 3**. A list of the DEGs in the cluster one.

| Gene ID | Gene | Sham | Stroke | Fold Change | p-value | Gene ID | Gene | Sham | Stroke | Fold Change | p-value |
|---|---|---|---|---|---|---|---|---|---|---|---|
| 18491 | Pappa | 0.120 | 0.51 | 4.1952 | 4E-16 | 12702 | Socs3 | 1.584 | 26.37 | 16.6560 | 8E-69 |
| 21463 | Tcp11 | 0.233 | 0.52 | 2.2342 | 3E-15 | 27279 | Tnfrsf12a | 3.022 | 27.24 | 9.0144 | 6E-60 |
| 11859 | Phox2a | 0.006 | 0.61 | 100.9033 | 2E-22 | 22695 | Zfp36 | 2.811 | 27.40 | 9.7469 | 1E-50 |
| 54140 | Avpr1a | 0.181 | 0.74 | 4.0804 | 2E-18 | 83397 | Akap12 | 7.775 | 29.32 | 3.7706 | 9E-24 |
| 69047 | Atp2c2 | 0.321 | 0.77 | 2.3910 | 1E-13 | 11504 | Adamts1 | 3.314 | 32.50 | 9.8049 | 1E-44 |
| 16399 | Itga2b | 0.331 | 0.82 | 2.4781 | 1E-21 | 13024 | Ctla2a | 5.574 | 36.73 | 6.5886 | 6E-46 |
| 232431 | Gprc5a | 0.146 | 0.93 | 6.3714 | 5E-13 | 12608 | Cebpb | 11.934 | 38.23 | 3.2031 | 7E-19 |
| 21679 | Tead4 | 0.343 | 0.95 | 2.7584 | 3E-29 | 12475 | Cd14 | 1.347 | 38.96 | 28.9299 | 7E-32 |
| 16373 | Irx3 | 0.099 | 1.15 | 11.5871 | 3E-14 | 18035 | Nfkbia | 20.167 | 45.52 | 2.2570 | 2E-20 |
| 20344 | Selp | 0.011 | 1.38 | 124.5033 | 2E-38 | 18263 | Odc1 | 19.296 | 54.42 | 2.8203 | 2E-29 |
| 20339 | Sele | 0.030 | 1.40 | 46.4031 | 2E-28 | 53324 | Nptx2 | 23.421 | 54.49 | 2.3267 | 4E-22 |
| 171530 | Ucn2 | 0.058 | 1.41 | 24.4330 | 2E-33 | 15901 | Id1 | 25.537 | 55.35 | 2.1674 | 4E-25 |
| 14064 | F2rl2 | 0.650 | 1.41 | 2.1734 | 2E-14 | 110454 | Ly6a | 24.956 | 59.37 | 2.3789 | 3E-23 |
| 74490 | Mamstr | 0.712 | 1.43 | 2.0157 | 2E-16 | 193740 | Hspa1a | 6.170 | 64.78 | 10.5001 | 8E-25 |
| 15227 | Foxf1 | 0.568 | 1.44 | 2.5317 | 7E-21 | 15368 | Hmox1 | 2.380 | 66.60 | 27.9848 | 2E-91 |
| 14161 | Fga | 0.009 | 1.53 | 168.3232 | 2E-22 | 76650 | Srxn1 | 32.061 | 68.86 | 2.1478 | 3E-21 |
| 18442 | P2ry2 | 0.208 | 1.60 | 7.6951 | 4E-31 | 12575 | Cdkn1a | 11.954 | 80.17 | 6.7066 | 9E-55 |
| 21926 | Tnf | 0.063 | 1.66 | 26.4737 | 2E-45 | 15511 | Hspa1b | 4.862 | 87.94 | 18.0876 | 1E-34 |
| 21950 | Tnfsf9 | 0.336 | 2.00 | 5.9527 | 8E-28 | 16819 | Lcn2 | 2.185 | 93.96 | 42.9936 | 1E-41 |
| 16878 | Lif | 0.197 | 2.34 | 11.8939 | 9E-36 | 15507 | Hspb1 | 12.856 | 143.20 | 11.1384 | 2E-48 |
| 329977 | Fhad1 | 1.155 | 2.34 | 2.0302 | 5E-17 | 17750 | Mt2 | 184.402 | 1698.87 | 9.2129 | 5E-43 |
| 14283 | Fosl1 | 0.025 | 2.60 | 103.2222 | 3E-32 | 17748 | Mt1 | 544.637 | 2279.70 | 4.1857 | 2E-34 |
| 72297 | B3gnt3 | 0.669 | 2.74 | 4.0915 | 6E-14 | 68278 | Ddx39 | 4.327 | 8.76 | 2.0248 | 5E-25 |
| 58801 | Pmaip1 | 1.085 | 3.06 | 2.8185 | 1E-17 | 18003 | Nedd9 | 2.839 | 5.76 | 2.0278 | 8E-20 |
| 104110 | Adcy4 | 1.461 | 3.12 | 2.1330 | 3E-18 | 213053 | Slc39a14 | 7.226 | 15.09 | 2.0876 | 1E-21 |
| 20310 | Cxcl2 | 0.030 | 3.43 | 115.4264 | 2E-15 | 20680 | Sox7 | 0.562 | 1.20 | 2.1426 | 8E-22 |
| 114332 | Lyve1 | 0.532 | 3.80 | 7.1508 | 6E-35 | 50493 | Txnrd1 | 16.670 | 37.27 | 2.2355 | 7E-25 |
| 101533 | Klk9 | 0.013 | 3.84 | 284.9056 | 6E-50 | 94092 | Trim16 | 0.525 | 1.17 | 2.2395 | 4E-29 |
| 75750 | Slc10a6 | 0.025 | 3.90 | 157.6564 | 5E-120 | 67941 | Rps27l | 26.034 | 58.48 | 2.2464 | 2E-21 |
| 18712 | Pim1 | 1.983 | 4.17 | 2.1038 | 2E-13 | 12654 | Chil1 | 3.840 | 8.68 | 2.2613 | 5E-18 |
| 13614 | Edn1 | 1.212 | 4.64 | 3.8230 | 3E-35 | 56200 | Ddx21 | 6.310 | 14.52 | 2.3011 | 1E-24 |
| 214855 | Arid5a | 1.908 | 4.70 | 2.4631 | 2E-14 | 80859 | Nfkbiz | 1.984 | 4.61 | 2.3256 | 3E-19 |
| 16193 | Il6 | 0.066 | 4.73 | 71.8821 | 4E-27 | 121022 | Mrps6 | 22.825 | 53.33 | 2.3366 | 5E-25 |
| 224405 | Cyyr1 | 1.722 | 5.20 | 3.0222 | 7E-35 | 12479 | Cd1d1 | 0.292 | 0.71 | 2.4225 | 2E-22 |
| 16156 | Il11 | 0.113 | 5.35 | 47.4396 | 4E-59 | 66540 | Fam107b | 2.214 | 5.39 | 2.4337 | 5E-23 |
| 14825 | Cxcl1 | 0.013 | 5.74 | 451.4273 | 5E-17 | 69660 | Tmbim1 | 11.143 | 27.64 | 2.4804 | 1E-28 |
| 72690 | Grrp1 | 2.773 | 5.89 | 2.1231 | 7E-31 | 13685 | Eif4ebp1 | 3.647 | 9.35 | 2.5643 | 3E-25 |
| 101401 | Adamts9 | 0.606 | 5.91 | 9.7511 | 5E-33 | 20848 | Stat3 | 14.475 | 43.17 | 2.9820 | 5E-41 |
| 67664 | Rnf125 | 0.824 | 6.12 | 7.4283 | 2E-64 | 17874 | Myd88 | 2.312 | 7.48 | 3.2351 | 4E-32 |
| 23886 | Gdf15 | 0.043 | 6.16 | 143.5938 | 5E-28 | 14528 | Gch1 | 0.432 | 1.45 | 3.3566 | 1E-18 |
| 20698 | Sphk1 | 1.625 | 7.28 | 4.4801 | 1E-25 | 108105 | B3gnt5 | 0.200 | 0.67 | 3.3694 | 4E-22 |
| 68895 | Rasl11a | 3.360 | 7.66 | 2.2791 | 3E-31 | 231805 | Pilra | 0.298 | 1.01 | 3.3915 | 2E-29 |
| 434215 | Lrrc32 | 1.578 | 7.70 | 4.8802 | 8E-13 | 14173 | Fgf2 | 1.575 | 5.43 | 3.4465 | 5E-32 |
| 16600 | Klf4 | 3.308 | 7.94 | 2.3997 | 4E-21 | 64540 | Tspan4 | 6.263 | 21.82 | 3.4833 | 9E-38 |
| 97165 | Hmgb2 | 0.896 | 8.14 | 9.0767 | 1E-45 | 414801 | Itprip | 0.340 | 1.31 | 3.8356 | 9E-16 |
| 53608 | Map3k6 | 1.845 | 8.18 | 4.4361 | 2E-78 | 16402 | Itga5 | 1.661 | 6.74 | 4.0592 | 7E-28 |
| 18787 | Serpine1 | 0.532 | 8.24 | 15.4729 | 2E-68 | 108052 | Slc14a1 | 2.372 | 9.68 | 4.0809 | 1E-59 |
| 98711 | Rdh10 | 3.009 | 8.64 | 2.8732 | 6E-21 | 216161 | Sbno2 | 3.115 | 14.34 | 4.6024 | 8E-50 |
| 19288 | Ptx3 | 0.096 | 9.03 | 93.7834 | 2E-49 | 78388 | Mvp | 2.366 | 11.37 | 4.8066 | 1E-43 |
| 18793 | Plaur | 0.547 | 10.28 | 18.8116 | 1E-24 | 235379 | Gldn | 0.285 | 1.49 | 5.2150 | 7E-33 |
| 69903 | Rasip1 | 4.265 | 10.54 | 2.4716 | 2E-14 | 77125 | Il33 | 9.001 | 48.07 | 5.3403 | 6E-25 |
| 226040 | Tmem252 | 0.819 | 10.93 | 13.3465 | 1E-46 | 19124 | Procr | 0.281 | 2.33 | 8.2686 | 3E-29 |
| 17133 | Maff | 1.778 | 11.12 | 6.2522 | 5E-45 | 12505 | Cd44 | 1.147 | 12.22 | 10.6513 | 2E-94 |
| 17135 | Mafk | 4.981 | 11.42 | 2.2922 | 8E-18 | 56708 | Clcf1 | 0.171 | 2.02 | 11.8173 | 5E-31 |
| 17112 | Tm4sf1 | 2.707 | 13.24 | 4.8907 | 6E-32 | 18414 | Osmr | 1.184 | 16.03 | 13.5375 | 1E-97 |
| 230784 | Sesn2 | 5.277 | 14.07 | 2.6663 | 3E-16 | 245527 | Eda2r | 0.049 | 0.68 | 13.9522 | 2E-28 |

© 2020. Wu J et al. Published online at http://www.aginganddisease.org/EN/10.14336/AD.2020.09206

| | | | | | | | | | | | |
|---|---|---|---|---|---|---|---|---|---|---|---|
| 57435 | Plin4 | 6.054 | 14.79 | 2.4430 | 3E-12 | 12768 | Ccr1 | 0.080 | 1.31 | 16.4396 | 8E-31 |
| 80837 | Rhoj | 1.354 | 15.13 | 11.1760 | 2E-98 | 68794 | Flnc | 0.612 | 10.30 | 16.8316 | 3E-91 |
| 15200 | Hbegf | 4.611 | 15.17 | 3.2908 | 5E-61 | 433470 | AA4671970 | .211 | 3.83 | 18.1478 | 6E-25 |
| 67951 | Tubb6 | 2.530 | 15.58 | 6.1571 | 5E-43 | 12051 | Bcl3 | 0.313 | 6.22 | 19.8648 | 2E-116 |
| 66282 | Tma16 | 6.202 | 15.63 | 2.5193 | 4E-21 | 18413 | Osm | 0.038 | 0.82 | 21.7043 | 1E-35 |
| 215418 | Csrnp1 | 6.792 | 15.68 | 2.3082 | 2E-12 | 56532 | Ripk3 | 0.036 | 0.77 | 21.7082 | 5E-46 |
| 18613 | Pecam1 | 6.776 | 15.70 | 2.3168 | 4E-22 | 20308 | Ccl9 | 0.272 | 6.94 | 25.5017 | 3E-41 |
| 20296 | Ccl2 | 0.068 | 15.83 | 231.6977 | 2E-29 | 11910 | Atf3 | 0.379 | 11.30 | 29.7684 | 2E-56 |
| 381823 | Apold1 | 2.131 | 15.97 | 7.4910 | 3E-21 | 14663 | Glycam1 | 0.046 | 1.46 | 31.5514 | 9E-23 |
| 15937 | Ier3 | 6.961 | 16.19 | 2.3255 | 3E-20 | 20293 | Ccl12 | 0.892 | 35.25 | 39.5282 | 4E-68 |
| 17872 | Ppp1r15a | 7.484 | 17.13 | 2.2883 | 1E-14 | 20292 | Ccl11 | 0.022 | 2.86 | 130.93264 | E-36 |
| 12609 | Cebpd | 3.511 | 21.84 | 6.2210 | 5E-49 | 16181 | Il1rn | 0.015 | 4.04 | 264.04873 | E-23 |

The expression levels of the genes in this cluster are increased by more than 2 folds with p-value < 0.05 at 1 day after stroke onset.

© 2020. Wu J et al. Published online at http://www.aginganddisease.org/EN/10.14336/AD.2020.09206

# SUPPLEMENTARY DATA

**Supplementary Table 4.** A list of the DEGs in the cluster two.

| Gene ID | Gene | Sham | Stroke | Fold Change | p_value | Gene ID | Gene | Sham | Stroke | Fold Change | p_value | Gene ID | Gene | Sham | Stroke | Fold Change | p_value |
|---|---|---|---|---|---|---|---|---|---|---|---|---|---|---|---|---|---|
| 217835 | Rin3 | 1.01 | 5.10 | 5.042 | 1E-26 | 20416 | Shc1 | 3.43 | 10.07 | 2.940 | 9E-21 | 22320 | Vamp8 | 6.81 | 23.38 | 3.432 | 2E-33 |
| 209200 | Dtx3l | 1.48 | 5.12 | 3.461 | 8E-36 | 27355 | Pald1 | 2.74 | 10.08 | 3.676 | 4E-18 | 20973 | Syngr2 | 4.85 | 23.42 | 4.833 | 4E-38 |
| 110095 | Pygl | 1.71 | 5.12 | 3.001 | 5E-16 | 14247 | Fli1 | 2.15 | 10.08 | 4.682 | 1E-28 | 67268 | Myl12a | 9.11 | 23.53 | 2.582 | 6E-23 |
| 78816 | Gmip | 1.63 | 5.17 | 3.168 | 4E-19 | 234779 | Plcg2 | 1.09 | 10.09 | 9.229 | 3E-36 | 18024 | Nfe2l2 | 5.57 | 23.70 | 4.255 | 4E-26 |
| 109901 | Cela1 | 2.12 | 5.21 | 2.461 | 5E-47 | 18950 | Pnp | 4.01 | 10.10 | 2.516 | 1E-18 | 21803 | Tgfb1 | 3.18 | 23.70 | 7.456 | 9E-39 |
| 107321 | Lpxn | 0.53 | 5.25 | 9.954 | 9E-63 | 52377 | Rcn3 | 2.61 | 10.10 | 3.871 | 2E-22 | 192176 | Flna | 8.83 | 23.92 | 2.709 | 9E-26 |
| 80879 | Slc16a3 | 1.75 | 5.26 | 2.998 | 7E-16 | 17970 | Ncf2 | 1.16 | 10.11 | 8.704 | 3E-42 | 12508 | Cd53 | 2.42 | 24.08 | 9.962 | 6E-40 |
| 18605 | Enpp1 | 0.92 | 5.27 | 5.760 | 1E-22 | 11810 | Apobec1 | 0.50 | 10.16 | 20.294 | 6E-48 | 71665 | Fuca1 | 9.70 | 24.22 | 2.497 | 3E-14 |
| 77057 | Ston1 | 1.91 | 5.28 | 2.766 | 4E-15 | 76408 | Abcc3 | 0.59 | 10.22 | 17.412 | 2E-33 | 21937 | Tnfrsf1a | 4.12 | 24.22 | 5.880 | 2E-66 |
| 11513 | Adcy7 | 1.51 | 5.30 | 3.514 | 2E-22 | 75547 | Akap13 | 3.74 | 10.27 | 2.742 | 2E-18 | 105855 | Nckap1l | 2.54 | 24.25 | 9.543 | 7E-44 |
| 217333 | Trim47 | 2.03 | 5.32 | 2.622 | 1E-20 | 15446 | Hpgd | 3.17 | 10.34 | 3.259 | 8E-20 | 19144 | Klk6 | 1.59 | 24.28 | 15.246 | 8E-73 |
| 320484 | Rasal3 | 0.56 | 5.33 | 9.536 | 4E-33 | 68195 | Rnaset2b | 3.91 | 10.38 | 2.656 | 8E-17 | 18822 | Plod1 | 5.36 | 24.51 | 4.576 | 4E-22 |
| 207839 | Galnt6 | 2.19 | 5.39 | 2.463 | 2E-20 | 22324 | Vav1 | 0.92 | 10.40 | 11.247 | 7E-48 | 18301 | Fxyd5 | 4.82 | 25.19 | 5.223 | 2E-27 |
| 17095 | Lyl1 | 1.12 | 5.39 | 4.821 | 2E-16 | 11727 | Ang | 1.39 | 10.41 | 7.475 | 1E-17 | 105348 | Golm1 | 5.55 | 25.38 | 4.574 | 8E-23 |
| 17110 | Lyz1 | 0.06 | 5.42 | 90.623 | 3E-52 | 17060 | Blnk | 2.77 | 10.43 | 3.761 | 1E-38 | 16331 | Inpp5d | 3.34 | 25.55 | 7.655 | 6E-32 |
| 229595 | Adamtsl4 | 1.64 | 5.43 | 3.310 | 5E-18 | 16985 | Lsp1 | 3.15 | 10.46 | 3.321 | 4E-25 | 66395 | Ahnak | 10.35 | 25.61 | 2.474 | 1E-24 |
| 16188 | Il3ra | 0.52 | 5.45 | 10.495 | 2E-28 | 16456 | F11r | 2.37 | 10.51 | 4.436 | 6E-31 | 12257 | Tspo | 4.62 | 25.63 | 5.546 | 3E-47 |
| 16453 | Jak3 | 1.66 | 5.46 | 3.286 | 5E-23 | 14595 | B4galt1 | 1.47 | 10.58 | 7.193 | 8E-47 | 26362 | Axl | 11.17 | 26.05 | 2.332 | 7E-24 |
| 72333 | Palld | 1.55 | 5.46 | 3.529 | 4E-21 | 70719 | Hmha1 | 1.16 | 10.75 | 9.258 | 4E-36 | 12267 | C3ar1 | 0.78 | 26.12 | 33.686 | 8E-62 |
| 217069 | Trim25 | 1.45 | 5.46 | 3.772 | 6E-35 | 71929 | Tmem123 | 3.69 | 10.76 | 2.919 | 2E-22 | 27419 | Naglu | 3.77 | 26.25 | 6.963 | 1E-24 |
| 212032 | Hk3 | 0.37 | 5.59 | 15.023 | 7E-64 | 50917 | Galns | 3.06 | 10.92 | 3.565 | 2E-16 | 12332 | Capg | 1.65 | 26.62 | 16.126 | 2E-56 |
| 50706 | Postn | 0.68 | 5.61 | 8.242 | 2E-26 | 11813 | Apoc2 | 0.06 | 10.95 | 186.840 | 4E-46 | 12045 | Bcl2a1b | 0.87 | 26.65 | 30.675 | 1E-72 |
| 67896 | Ccdc80 | 2.45 | 5.64 | 2.303 | 4E-21 | 68480 | 1110007C09Rik | 3.71 | 10.97 | 2.955 | 6E-26 | 24044 | Scamp2 | 7.44 | 26.79 | 3.600 | 9E-27 |
| 15223 | Foxj1 | 1.35 | 5.64 | 4.185 | 2E-19 | 240168 | Rasgrp3 | 2.71 | 11.00 | 4.053 | 1E-15 | 12827 | Col4a2 | 8.00 | 26.82 | 3.350 | 1E-28 |
| 69774 | Ms4a6b | 0.59 | 5.67 | 9.616 | 9E-53 | 94176 | Dock2 | 0.80 | 11.01 | 13.809 | 3E-45 | 67448 | Plxdc2 | 10.89 | 27.58 | 2.532 | 9E-18 |
| 22325 | Vav2 | 2.40 | 5.68 | 2.369 | 1E-18 | 329679 | Fnip2 | 4.89 | 11.02 | 2.251 | 8E-17 | 104759 | Pld4 | 2.87 | 27.58 | 9.597 | 6E-35 |
| 21815 | Tgif1 | 0.64 | 5.69 | 8.901 | 8E-46 | 20302 | Ccl3 | 0.10 | 11.23 | 116.447 | 4E-117 | 16859 | Lgals9 | 3.62 | 27.71 | 7.652 | 6E-50 |
| 80287 | Apobec3 | 1.08 | 5.71 | 5.285 | 2E-49 | 17000 | Ltbr | 3.73 | 11.24 | 3.014 | 1E-37 | 17969 | Ncf1 | 2.47 | 28.32 | 11.468 | 1E-42 |
| 26382 | Fgd2 | 1.45 | 5.71 | 3.937 | 3E-21 | 106512 | Gpsm3 | 1.49 | 11.27 | 7.541 | 9E-31 | 14017 | Evi2a | 10.51 | 28.34 | 2.696 | 9E-16 |
| 13709 | Elf1 | 2.11 | 5.75 | 2.723 | 5E-19 | 67844 | Rab32 | 0.67 | 11.28 | 16.733 | 1E-33 | 67893 | Tmem86a | 8.18 | 29.03 | 3.547 | 1E-24 |
| 330217 | Gal3st4 | 1.30 | 5.76 | 4.417 | 2E-16 | 20469 | Sipa1 | 3.34 | 11.36 | 3.397 | 1E-20 | 15976 | Ifnar2 | 13.19 | 29.31 | 2.222 | 5E-22 |
| 394432 | Ugt1a7c | 0.31 | 5.77 | 18.761 | 7E-52 | 69710 | Arap1 | 3.49 | 11.39 | 3.264 | 1E-20 | 326618 | Tpm4 | 10.03 | 29.78 | 2.968 | 2E-31 |
| 14676 | Gna15 | 0.87 | 5.78 | 6.626 | 1E-35 | 268396 | Sh3pxd2b | 3.92 | 11.39 | 2.905 | 3E-26 | 66147 | Necap2 | 10.35 | 29.85 | 2.886 | 6E-22 |
| 74055 | Plce1 | 2.08 | 5.80 | 2.789 | 6E-46 | 18129 | Notch2 | 4.83 | 11.43 | 2.367 | 7E-18 | 13618 | Ednrb | 13.77 | 29.93 | 2.174 | 4E-30 |
| 15162 | Hck | 0.75 | 5.82 | 7.804 | 8E-41 | 11491 | Adam17 | 2.96 | 11.44 | 3.863 | 1E-16 | 66058 | Tmem176a | 8.67 | 29.94 | 3.454 | 1E-22 |
| 23900 | Hcst | 1.03 | 5.82 | 5.670 | 1E-26 | 76281 | Tax1bp3 | 4.16 | 11.50 | 2.761 | 6E-25 | 68420 | Ankrd13a | 13.40 | 30.05 | 2.243 | 3E-16 |
| 72512 | Tmem173 | 1.17 | 5.85 | 5.008 | 4E-39 | 243912 | Hspb6 | 3.66 | 11.54 | 3.151 | 3E-42 | 13733 | Adgre1 | 1.69 | 30.21 | 17.873 | 1E-34 |
| 74202 | Fblim1 | 0.38 | 5.87 | 15.303 | 1E-47 | 233571 | P2ry6 | 1.17 | 11.58 | 9.922 | 2E-44 | 74048 | 4632428N05Rik | 5.15 | 30.36 | 5.889 | 4E-28 |
| 21934 | Tnfrsf11a | 1.76 | 5.87 | 3.344 | 3E-16 | 67880 | Dcxr | 4.58 | 11.70 | 2.554 | 6E-19 | 20397 | Sgpl1 | 8.61 | 30.66 | 3.561 | 9E-31 |

© 2020. Wu J et al. Published online at http://www.aginganddisease.org/EN/10.14336/AD.2020.09206

# SUPPLEMENTARY DATA

| | | | | | | | | | | | | | | | | | |
|---|---|---|---|---|---|---|---|---|---|---|---|---|---|---|---|---|---|
| 21676 | Tead1 | 2.65 | 5.89 | 2.223 | 2E-28 | 27056 | Irf5 | 1.48 | 11.74 | 7.944 | 1E-47 | 12192 | Zfp36l1 | 10.96 | 30.77 | 2.807 | 2E-24 |
| 19253 | Ptpn18 | 0.71 | 5.95 | 8.367 | 4E-41 | 19703 | Renbp | 1.85 | 11.76 | 6.362 | 3E-37 | 11853 | Rhoc | 8.76 | 30.95 | 3.534 | 3E-32 |
| 107885 | Mthfs | 2.54 | 5.99 | 2.355 | 1E-17 | 14792 | Lpcat3 | 4.88 | 11.83 | 2.426 | 9E-17 | 21346 | Tagln2 | 6.57 | 31.07 | 4.732 | 2E-44 |
| 19106 | Eif2ak2 | 1.87 | 5.99 | 3.202 | 3E-24 | 279572 | Tlr13 | 0.58 | 11.89 | 20.638 | 6E-48 | 12606 | Cebpa | 5.16 | 31.07 | 6.024 | 8E-43 |
| 66895 | Pxdc1 | 2.18 | 6.02 | 2.767 | 6E-20 | 74211 | 1700017B05Rik | 3.74 | 11.90 | 3.180 | 2E-21 | 16889 | Lipa | 5.34 | 31.49 | 5.902 | 2E-22 |
| 16194 | Il6ra | 1.50 | 6.04 | 4.037 | 7E-42 | 67776 | Vwa5a | 4.56 | 12.15 | 2.668 | 3E-25 | 17079 | Cd180 | 0.88 | 32.33 | 36.837 | 8E-49 |
| 14537 | Gcnt1 | 0.66 | 6.05 | 9.234 | 3E-20 | 19303 | Pxn | 5.66 | 12.20 | 2.155 | 3E-22 | 16414 | Itgb2 | 1.52 | 32.47 | 21.328 | 1E-55 |
| 97165 | Hmgb2 | 1.46 | 6.06 | 4.143 | 2E-29 | 71279 | Slc29a3 | 3.53 | 12.21 | 3.460 | 2E-21 | 13430 | Dnm2 | 13.32 | 32.54 | 2.443 | 1E-14 |
| 30955 | Pik3cg | 0.78 | 6.08 | 7.780 | 3E-26 | 18792 | Plau | 1.45 | 12.24 | 8.445 | 1E-34 | 11745 | Anxa3 | 4.45 | 32.55 | 7.321 | 1E-32 |
| 14159 | Fes | 0.85 | 6.09 | 7.200 | 3E-44 | 11717 | Ampd3 | 5.86 | 12.24 | 2.089 | 1E-21 | 21894 | Tln1 | 7.92 | 32.56 | 4.113 | 2E-24 |
| 78558 | Htra3 | 1.39 | 6.12 | 4.399 | 1E-18 | 216991 | Adap2 | 2.13 | 12.34 | 5.799 | 2E-26 | 18826 | Lcp1 | 2.38 | 32.94 | 13.866 | 2E-47 |
| 17215 | Mcm3 | 0.86 | 6.15 | 7.127 | 1E-28 | 69769 | Tnfaip8l2 | 1.45 | 12.41 | 8.534 | 7E-49 | 20345 | Selplg | 9.64 | 33.48 | 3.473 | 5E-17 |
| 226652 | Arhgap30 | 0.84 | 6.21 | 7.367 | 7E-37 | 13830 | Stom | 4.27 | 12.43 | 2.910 | 9E-21 | 81910 | Rrbp1 | 10.91 | 33.99 | 3.114 | 2E-21 |
| 67689 | Aldh3b1 | 2.02 | 6.27 | 3.101 | 2E-15 | 171285 | Havcr2 | 1.12 | 12.53 | 11.157 | 2E-27 | 65972 | Ifi30 | 2.77 | 34.05 | 12.282 | 4E-52 |
| 20963 | Syk | 0.73 | 6.27 | 8.637 | 2E-34 | 23880 | Fyb | 0.89 | 12.53 | 14.145 | 3E-47 | 52668 | Ifi27 | 12.73 | 34.41 | 2.703 | 5E-20 |
| 320487 | Heatr5a | 1.91 | 6.29 | 3.289 | 1E-16 | 18753 | Prkcd | 3.22 | 12.62 | 3.924 | 4E-16 | 17160 | Man2b2 | 10.63 | 35.07 | 3.301 | 7E-15 |
| 11593 | Aga | 2.81 | 6.30 | 2.240 | 2E-19 | 22323 | Vasp | 5.23 | 12.77 | 2.441 | 4E-18 | 11629 | Aif1 | 4.89 | 35.15 | 7.182 | 7E-42 |
| 23921 | Sh2b2 | 2.86 | 6.31 | 2.203 | 6E-26 | 21391 | Tbxas1 | 0.72 | 12.79 | 17.794 | 7E-39 | 16195 | Il6st | 17.39 | 35.18 | 2.023 | 1E-20 |
| 100169 | Phactr4 | 3.14 | 6.42 | 2.045 | 3E-19 | 17916 | Myo1f | 0.61 | 12.82 | 20.939 | 7E-42 | 21813 | Tgfbr2 | 4.71 | 36.40 | 7.729 | 5E-36 |
| 259300 | Ehd2 | 2.56 | 6.44 | 2.517 | 7E-25 | 52855 | Lair1 | 2.23 | 12.92 | 5.796 | 2E-28 | 23890 | Gpr34 | 7.74 | 36.42 | 4.704 | 9E-15 |
| 18018 | Nfatc1 | 1.82 | 6.50 | 3.570 | 1E-24 | 108101 | Fermt3 | 1.34 | 12.98 | 9.709 | 5E-49 | 11857 | Arhgdib | 7.58 | 36.88 | 4.868 | 3E-28 |
| 12830 | Col4a5 | 0.72 | 6.51 | 9.078 | 8E-24 | 270084 | Lpcat2 | 3.27 | 13.02 | 3.981 | 3E-27 | 319939 | Tns3 | 10.36 | 37.69 | 3.638 | 4E-17 |
| 24055 | Sh3bp2 | 0.71 | 6.60 | 9.243 | 3E-46 | 223453 | Dap | 3.71 | 13.13 | 3.544 | 2E-26 | 12826 | Col4a1 | 6.14 | 37.83 | 6.159 | 4E-41 |
| 235406 | Snx33 | 2.63 | 6.61 | 2.515 | 1E-17 | 211286 | Cln5 | 5.22 | 13.16 | 2.521 | 2E-18 | 18858 | Pmp22 | 10.34 | 38.06 | 3.681 | 2E-32 |
| 80898 | Erap1 | 2.78 | 6.62 | 2.377 | 2E-23 | 18542 | Pcolce | 4.51 | 13.34 | 2.958 | 5E-19 | 21356 | Tapbp | 13.18 | 38.68 | 2.934 | 7E-27 |
| 78781 | Zc3hav1 | 1.57 | 6.67 | 4.248 | 4E-50 | 227696 | Phyhd1 | 4.36 | 13.50 | 3.094 | 1E-18 | 56193 | Plek | 3.76 | 38.71 | 10.290 | 2E-38 |
| 52009 | Hn1l | 1.62 | 6.67 | 4.110 | 2E-24 | 170768 | Pfkfb3 | 6.48 | 13.74 | 2.121 | 5E-18 | 16412 | Itgb1 | 16.07 | 39.16 | 2.437 | 4E-19 |
| 102545 | Cmtm7 | 1.33 | 6.74 | 5.055 | 7E-27 | 73656 | Ms4a6c | 0.46 | 13.79 | 30.289 | 2E-60 | 433375 | Creg1 | 11.38 | 39.57 | 3.478 | 1E-20 |
| 67742 | Samsn1 | 0.69 | 6.76 | 9.827 | 5E-39 | 27060 | Tcirg1 | 2.52 | 14.16 | 5.628 | 2E-37 | 20148 | Dhrs3 | 9.27 | 39.78 | 4.290 | 4E-19 |
| 100952 | Emilin1 | 0.83 | 6.77 | 8.135 | 2E-24 | 54325 | Elovl1 | 4.45 | 14.22 | 3.193 | 1E-22 | 16658 | Mafb | 7.66 | 40.08 | 5.235 | 3E-28 |
| 73690 | Glipr1 | 0.68 | 6.81 | 10.035 | 1E-24 | 13423 | Dnase2a | 1.87 | 14.22 | 7.593 | 5E-35 | 16409 | Itgam | 4.47 | 40.19 | 8.984 | 5E-41 |
| 12832 | Col5a2 | 0.60 | 6.84 | 11.369 | 2E-18 | 170743 | Tlr7 | 1.03 | 14.30 | 13.916 | 1E-48 | 12305 | Ddr1 | 19.50 | 40.26 | 2.065 | 1E-12 |
| 100017 | Ldlrap1 | 1.29 | 6.87 | 5.324 | 3E-29 | 67457 | Frmd8 | 5.05 | 14.55 | 2.879 | 3E-15 | 114584 | Clic1 | 6.21 | 40.92 | 6.591 | 3E-48 |
| 21425 | Tfeb | 3.42 | 6.89 | 2.014 | 1E-16 | 14728 | Lilrb4a | 0.11 | 14.61 | 129.066 | 1E-84 | 100034251 | Wfdc17 | 0.70 | 41.09 | 58.371 | 9E-42 |
| 16543 | Mdfic | 0.94 | 6.90 | 7.349 | 2E-27 | 23943 | Esyt1 | 4.17 | 14.93 | 3.579 | 8E-18 | 21452 | Tcn2 | 7.41 | 41.82 | 5.645 | 2E-23 |
| 74096 | Hvcn1 | 0.73 | 6.92 | 9.487 | 6E-31 | 22436 | Xdh | 2.40 | 15.07 | 6.288 | 6E-23 | 29876 | Clic4 | 19.53 | 41.92 | 2.147 | 8E-21 |
| 14594 | Ggta1 | 1.29 | 6.95 | 5.389 | 2E-43 | 65221 | Slc15a3 | 0.60 | 15.17 | 25.102 | 9E-59 | 140570 | Plxnb2 | 12.72 | 43.13 | 3.392 | 3E-17 |
| 16190 | Il4ra | 1.21 | 6.98 | 5.746 | 7E-62 | 11690 | Alox5ap | 2.56 | 15.18 | 5.940 | 3E-27 | 54445 | Unc93b1 | 5.26 | 43.73 | 8.308 | 8E-45 |
| 224109 | Nrros | 1.67 | 7.04 | 4.227 | 2E-24 | 83768 | Dpp7 | 4.95 | 15.18 | 3.065 | 3E-17 | 12111 | Bgn | 6.32 | 44.45 | 7.035 | 7E-23 |
| 17390 | Mmp2 | 0.79 | 7.06 | 8.880 | 2E-30 | 54353 | Skap2 | 5.21 | 15.19 | 2.912 | 1E-19 | 30791 | Slc39a1 | 21.37 | 45.48 | 2.128 | 1E-21 |
| 16822 | Lcp2 | 0.77 | 7.10 | 9.220 | 1E-40 | 74191 | P2ry13 | 4.54 | 15.21 | 3.350 | 2E-14 | 65963 | Tmem176b | 16.08 | 45.50 | 2.830 | 2E-23 |
| 16154 | Il10ra | 1.53 | 7.17 | 4.699 | 5E-39 | 76448 | Ppp1r18 | 3.09 | 15.40 | 4.991 | 3E-37 | 11801 | Cd5l | 0.01 | 45.60 | 4514.420 | 7E-49 |
| 232201 | Arhgap25 | 2.53 | 7.18 | 2.837 | 1E-18 | 98878 | Ehd4 | 4.62 | 15.47 | 3.349 | 6E-35 | 11974 | Atp6v0e | 20.87 | 47.37 | 2.270 | 1E-16 |
| 22778 | Ikzf1 | 0.92 | 7.19 | 7.814 | 1E-35 | 58859 | Efemp2 | 5.04 | 15.58 | 3.088 | 6E-23 | 58809 | Rnase4 | 7.73 | 49.05 | 6.348 | 4E-18 |
| 16773 | Lama2 | 3.10 | 7.22 | 2.325 | 4E-15 | 320404 | Itpkb | 6.11 | 15.60 | 2.553 | 1E-15 | 17159 | Man2b1 | 10.77 | 49.44 | 4.589 | 9E-27 |

| | | | | | | | | | | | | | | | | | |
|---|---|---|---|---|---|---|---|---|---|---|---|---|---|---|---|---|---|
| 217262 | Abca9 | 1.98 | 7.22 | 3.645 | 2E-20 | 83490 | Pik3ap1 | 1.13 | 15.65 | 13.879 | 2E-54 | 56212 | Rhog | 16.30 | 51.75 | 3.175 | 8E-18 |
| 20540 | Slc7a7 | 0.72 | 7.23 | 10.061 | 9E-37 | 74646 | Spsb1 | 6.16 | 15.79 | 2.563 | 6E-21 | 12843 | Col1a2 | 4.74 | 53.08 | 11.192 | 3E-19 |
| 70021 | Nt5dc2 | 1.67 | 7.39 | 4.412 | 2E-18 | 17132 | Maf | 6.97 | 15.83 | 2.272 | 2E-16 | 192187 | Stab1 | 2.64 | 53.30 | 20.195 | 1E-24 |
| 18439 | P2rx7 | 2.32 | 7.52 | 3.237 | 4E-26 | 218454 | Lhfpl2 | 3.57 | 15.86 | 4.444 | 7E-22 | 13036 | Ctsh | 4.49 | 53.35 | 11.878 | 2E-43 |
| 319622 | Itpripl2 | 1.79 | 7.57 | 4.222 | 8E-31 | 74091 | Npl | 3.08 | 16.00 | 5.194 | 2E-24 | 12825 | Col3a1 | 0.76 | 53.75 | 70.818 | 4E-33 |
| 226594 | Rcsd1 | 2.22 | 7.58 | 3.412 | 9E-22 | 18073 | Nid1 | 1.71 | 16.19 | 9.494 | 7E-33 | 106572 | Rab31 | 21.28 | 54.17 | 2.546 | 6E-18 |
| 66824 | Pycard | 1.36 | 7.62 | 5.590 | 9E-44 | 215280 | Wipf1 | 3.57 | 16.29 | 4.565 | 2E-24 | 12268 | C4b | 5.04 | 55.26 | 10.960 | 3E-80 |
| 17085 | Ly9 | 0.08 | 7.63 | 94.114 | 2E-54 | 12521 | Cd82 | 5.68 | 16.50 | 2.905 | 3E-17 | 20229 | Sat1 | 21.70 | 55.43 | 2.555 | 1E-23 |
| 59010 | Sqrdl | 2.59 | 7.64 | 2.945 | 2E-19 | 59126 | Nek6 | 8.07 | 16.56 | 2.052 | 1E-27 | 14268 | Fn1 | 12.97 | 56.17 | 4.332 | 5E-16 |
| 102294 | Cyp4v3 | 2.53 | 7.67 | 3.027 | 4E-30 | 13601 | Ecm1 | 3.25 | 16.58 | 5.097 | 5E-19 | 17698 | Msn | 7.53 | 57.03 | 7.574 | 1E-59 |
| 268301 | Sowahc | 3.00 | 7.71 | 2.566 | 1E-20 | 58198 | Sall1 | 4.82 | 16.61 | 3.449 | 9E-21 | 11829 | Aqp4 | 21.66 | 57.08 | 2.635 | 6E-30 |
| 19354 | Rac2 | 0.52 | 7.73 | 14.820 | 5E-52 | 21810 | Tgfbi | 1.11 | 16.71 | 15.060 | 5E-54 | 67963 | Npc2 | 12.39 | 57.30 | 4.624 | 7E-32 |
| 107581 | Col16a1 | 2.36 | 7.75 | 3.284 | 2E-15 | 20111 | Rps6ka1 | 5.55 | 16.77 | 3.021 | 7E-18 | 11747 | Anxa5 | 22.60 | 57.56 | 2.548 | 7E-18 |
| 226527 | BC026585 | 2.60 | 7.76 | 2.980 | 3E-18 | 26433 | Plod3 | 8.00 | 16.81 | 2.102 | 2E-18 | 11303 | Abca1 | 7.85 | 59.26 | 7.552 | 6E-45 |
| 226421 | Rab7b | 0.48 | 7.76 | 16.053 | 2E-56 | 13132 | Dab2 | 2.49 | 16.95 | 6.806 | 9E-43 | 11867 | Arpc1b | 6.00 | 59.42 | 9.903 | 3E-57 |
| 17219 | Mcm6 | 2.87 | 7.77 | 2.711 | 9E-19 | 67203 | Nde1 | 5.87 | 17.06 | 2.906 | 1E-15 | 14131 | Fcgr3 | 5.93 | 59.59 | 10.052 | 4E-67 |
| 13713 | Elk3 | 2.54 | 7.84 | 3.085 | 4E-25 | 74760 | Rab3il1 | 3.94 | 17.09 | 4.338 | 2E-28 | 67865 | Rgs10 | 24.93 | 61.02 | 2.447 | 2E-22 |
| 98365 | Slamf9 | 0.70 | 7.84 | 11.163 | 2E-54 | 15163 | Hcls1 | 1.70 | 17.17 | 10.108 | 2E-42 | 56356 | Gltp | 15.25 | 63.58 | 4.168 | 8E-21 |
| 20491 | Sla | 3.03 | 7.85 | 2.594 | 1E-22 | 30794 | Pdlim4 | 5.80 | 17.18 | 2.960 | 3E-31 | 71994 | Cnn3 | 26.46 | 66.86 | 2.526 | 2E-43 |
| 15216 | Hfe | 1.86 | 7.89 | 4.244 | 2E-28 | 13732 | Emp3 | 2.64 | 17.31 | 6.546 | 6E-29 | 12842 | Col1a1 | 2.73 | 68.95 | 25.289 | 9E-20 |
| 16949 | Loxl1 | 0.77 | 7.89 | 10.237 | 1E-21 | 140780 | Bmp2k | 4.98 | 17.44 | 3.499 | 6E-20 | 11520 | Plin2 | 2.94 | 69.28 | 23.601 | 2E-41 |
| 52150 | Kcnk6 | 1.34 | 7.91 | 5.903 | 8E-30 | 209378 | Itih5 | 6.22 | 17.44 | 2.803 | 2E-20 | 110006 | Gusb | 4.47 | 71.90 | 16.097 | 1E-33 |
| 14276 | Folr2 | 0.41 | 7.92 | 19.558 | 2E-28 | 29875 | Iqgap1 | 4.88 | 17.45 | 3.577 | 4E-18 | 16002 | Igf2 | 14.08 | 77.47 | 5.500 | 2E-25 |
| 12752 | Cln3 | 2.99 | 8.01 | 2.676 | 5E-16 | 16403 | Itga6 | 5.12 | 17.46 | 3.408 | 2E-27 | 20971 | Sdc4 | 35.03 | 81.13 | 2.316 | 3E-27 |
| 74761 | Mxra8 | 1.81 | 8.02 | 4.428 | 2E-28 | 16000 | Igf1 | 1.81 | 17.57 | 9.708 | 6E-13 | 16784 | Lamp2 | 33.17 | 81.18 | 2.447 | 7E-19 |
| 68612 | Ube2c | 0.22 | 8.07 | 37.414 | 4E-19 | 67213 | Cmtm6 | 8.16 | 17.67 | 2.164 | 5E-20 | 13051 | Cx3cr1 | 12.46 | 83.25 | 6.683 | 2E-25 |
| 66240 | Kcne1l | 2.18 | 8.16 | 3.742 | 1E-17 | 218203 | Mylip | 5.61 | 18.24 | 3.252 | 3E-20 | 13057 | Cyba | 6.81 | 86.86 | 12.761 | 3E-61 |
| 269823 | Pon3 | 0.82 | 8.18 | 10.013 | 4E-22 | 72318 | Cyth4 | 2.41 | 18.34 | 7.612 | 3E-48 | 16419 | Itgb5 | 11.90 | 88.23 | 7.415 | 6E-26 |
| 12273 | C5ar1 | 0.28 | 8.21 | 29.677 | 1E-58 | 20409 | Ostf1 | 5.59 | 18.43 | 3.295 | 3E-27 | 56188 | Fxyd1 | 36.00 | 94.05 | 2.613 | 6E-21 |
| 239743 | Klhl6 | 0.68 | 8.26 | 12.104 | 5E-40 | 140497 | AF251705 | 1.37 | 18.44 | 13.431 | 3E-56 | 99543 | Olfml3 | 9.20 | 94.82 | 10.302 | 9E-32 |
| 109305 | Orai1 | 3.38 | 8.27 | 2.443 | 5E-15 | 80888 | Hspb8 | 6.86 | 18.57 | 2.706 | 2E-30 | 76960 | Bcas1 | 37.92 | 97.20 | 2.563 | 5E-15 |
| 21973 | Top2a | 0.25 | 8.37 | 33.197 | 2E-19 | 68119 | Cmtm3 | 3.75 | 18.69 | 4.985 | 3E-28 | 75612 | Gns | 32.71 | 97.96 | 2.995 | 2E-17 |
| 219144 | Arl11 | 0.36 | 8.39 | 23.092 | 1E-40 | 16155 | Il10rb | 4.17 | 18.80 | 4.512 | 3E-26 | 16011 | Igfbp5 | 40.99 | 98.99 | 2.415 | 5E-30 |
| 243771 | Parp12 | 2.81 | 8.44 | 3.002 | 3E-40 | 80281 | Cttnbp2nl | 8.27 | 18.83 | 2.276 | 3E-18 | 16852 | Lgals1 | 16.05 | 101.30 | 6.311 | 6E-24 |
| 76088 | Dock8 | 0.85 | 8.51 | 9.985 | 4E-34 | 12822 | Col18a1 | 0.99 | 18.97 | 19.213 | 2E-29 | 19025 | Ctsa | 23.35 | 101.62 | 4.352 | 1E-23 |
| 54486 | Hpgds | 1.71 | 8.60 | 5.041 | 8E-15 | 15979 | Ifngr1 | 6.90 | 18.98 | 2.750 | 5E-36 | 52585 | Dhrs1 | 32.55 | 101.84 | 3.128 | 3E-20 |
| 217203 | Tmem106a | 0.35 | 8.63 | 24.959 | 2E-56 | 71602 | Myo1e | 5.08 | 19.14 | 3.767 | 3E-24 | 17084 | Ly86 | 7.08 | 108.91 | 15.388 | 3E-56 |
| 22146 | Tuba1c | 0.68 | 8.71 | 12.724 | 3E-54 | 17387 | Mmp14 | 3.92 | 19.16 | 4.888 | 5E-25 | 16008 | Igfbp2 | 24.55 | 109.05 | 4.443 | 2E-16 |
| 64099 | Parvg | 1.60 | 8.73 | 5.449 | 2E-27 | 22329 | Vcam1 | 8.12 | 19.47 | 2.399 | 1E-29 | 72042 | Cotl1 | 28.66 | 120.41 | 4.202 | 2E-21 |
| 11501 | Adam8 | 0.26 | 8.74 | 33.674 | 2E-67 | 54519 | Apbb1ip | 2.63 | 19.52 | 7.411 | 9E-43 | 83433 | Trem2 | 7.07 | 128.79 | 18.215 | 3E-50 |
| 15277 | Hk2 | 0.91 | 8.74 | 9.576 | 1E-49 | 14726 | Pdpn | 5.16 | 19.59 | 3.799 | 1E-45 | 14707 | Gng5 | 46.82 | 128.80 | 2.751 | 3E-22 |
| 56857 | Slc37a2 | 0.83 | 8.79 | 10.627 | 7E-35 | 101488 | Slco2b1 | 3.75 | 19.59 | 5.226 | 5E-27 | 20716 | Serpina3n | 4.91 | 130.97 | 26.697 | 1E-181 |
| 56693 | Crtap | 2.74 | 8.81 | 3.217 | 4E-21 | 74645 | Fam46c | 0.68 | 19.79 | 29.233 | 2E-31 | 16792 | Laptm5 | 12.96 | 132.27 | 10.204 | 8E-45 |
| 56743 | Lat2 | 1.07 | 8.96 | 8.352 | 1E-66 | 19128 | Pros1 | 3.71 | 19.89 | 5.362 | 2E-55 | 15211 | Hexa | 21.36 | 136.55 | 6.391 | 2E-24 |
| 12489 | Cd33 | 1.66 | 9.00 | 5.419 | 2E-31 | 19205 | Ptbp1 | 8.50 | 19.93 | 2.345 | 3E-28 | 12978 | Csf1r | 24.73 | 143.25 | 5.793 | 4E-32 |
| 17355 | Aff1 | 4.29 | 9.08 | 2.119 | 1E-22 | 192193 | Edem1 | 5.64 | 20.10 | 3.564 | 2E-16 | 13039 | Ctsl | 33.45 | 144.96 | 4.333 | 1E-40 |
| 12642 | Ch25h | 0.69 | 9.10 | 13.274 | 6E-68 | 232157 | Mob1a | 7.52 | 20.15 | 2.678 | 2E-19 | 80891 | Fcrls | 9.74 | 154.62 | 15.881 | 3E-36 |

© 2020. Wu J et al. Published online at http://www.aginganddisease.org/EN/10.14336/AD.2020.09206

# SUPPLEMENTARY DATA

| | | | | | | | | | | | | | | | | | |
|---|---|---|---|---|---|---|---|---|---|---|---|---|---|---|---|---|---|
| 17183 | Matn4 | 2.53 | 9.15 | 3.619 | 4E-15 | 242687 | Wasf2 | 6.31 | 20.18 | 3.198 | 2E-22 | 22228 | Ucp2 | 12.09 | 157.20 | 13.000 | 2E-26 |
| 29810 | Bag3 | 3.42 | 9.19 | 2.691 | 2E-32 | 12977 | Csf1 | 5.71 | 20.34 | 3.563 | 4E-39 | 93695 | Gpnmb | 0.88 | 162.90 | 184.479 | 2E-50 |
| 13730 | Emp1 | 0.46 | 9.21 | 19.944 | 1E-54 | 22319 | Vamp3 | 9.88 | 20.43 | 2.069 | 7E-16 | 14127 | Fcer1g | 9.47 | 171.68 | 18.130 | 9E-70 |
| 16973 | Lrp5 | 2.75 | 9.22 | 3.348 | 5E-16 | 56722 | Litaf | 5.73 | 20.43 | 3.565 | 1E-37 | 12527 | Cd9 | 20.59 | 175.61 | 8.527 | 1E-56 |
| 20852 | Stat6 | 3.65 | 9.34 | 2.559 | 1E-17 | 232345 | A2m | 0.47 | 20.44 | 43.115 | 2E-84 | 11758 | Prdx6 | 72.04 | 181.91 | 2.525 | 7E-19 |
| 106878 | Smim3 | 2.86 | 9.36 | 3.272 | 1E-39 | 213019 | Pdlim2 | 5.14 | 20.45 | 3.977 | 7E-18 | 12514 | Cd68 | 7.11 | 182.78 | 25.711 | 5E-53 |
| 21825 | Thbs1 | 0.53 | 9.37 | 17.711 | 2E-26 | 74030 | Rin2 | 7.09 | 20.49 | 2.888 | 1E-15 | 12512 | Cd63 | 45.66 | 203.23 | 4.451 | 2E-46 |
| 12986 | Csf3r | 0.73 | 9.39 | 12.881 | 1E-43 | 66868 | Mfsd1 | 8.21 | 20.56 | 2.506 | 3E-18 | 17476 | Mpeg1 | 6.11 | 214.70 | 35.141 | 1E-47 |
| 17534 | Mrc2 | 2.24 | 9.39 | 4.189 | 3E-21 | 12523 | Cd84 | 0.91 | 20.57 | 22.573 | 6E-43 | 20692 | Sparc | 77.16 | 215.07 | 2.787 | 2E-20 |
| 12495 | Entpd1 | 3.32 | 9.40 | 2.832 | 4E-26 | 12493 | Cd37 | 2.02 | 20.66 | 10.220 | 1E-38 | 19141 | Lgmn | 51.76 | 264.04 | 5.101 | 3E-28 |
| 12633 | Cflar | 3.73 | 9.42 | 2.525 | 4E-16 | 21812 | Tgfbr1 | 5.55 | 20.74 | 3.741 | 2E-19 | 64138 | Ctsz | 21.51 | 273.93 | 12.736 | 3E-44 |
| 22379 | Fmnl3 | 3.07 | 9.43 | 3.068 | 1E-22 | 18791 | Plat | 10.03 | 21.06 | 2.100 | 2E-19 | 14824 | Grn | 22.53 | 313.43 | 13.913 | 2E-29 |
| 14747 | Cmklr1 | 1.07 | 9.58 | 8.947 | 4E-47 | 20375 | Spi1 | 2.37 | 21.08 | 8.907 | 2E-51 | 22352 | Vim | 18.85 | 326.08 | 17.302 | 1E-58 |
| 13040 | Ctss | 0.98 | 9.62 | 9.842 | 9E-50 | 17096 | Lyn | 2.71 | 21.39 | 7.882 | 9E-45 | 22041 | Trf | 97.94 | 366.56 | 3.743 | 7E-20 |
| 109225 | Ms4a7 | 0.18 | 9.70 | 54.916 | 2E-32 | 13032 | Ctsc | 2.27 | 21.55 | 9.486 | 6E-63 | 14580 | Gfap | 53.93 | 829.64 | 15.383 | 2E-89 |
| 228961 | Npepl1 | 4.91 | 9.84 | 2.004 | 6E-14 | 83924 | Gpr137b | 8.14 | 21.74 | 2.670 | 4E-16 | 20750 | Spp1 | 7.20 | 915.32 | 127.041 | 2E-49 |
| 16391 | Irf9 | 2.86 | 9.91 | 3.464 | 6E-39 | 12476 | Cd151 | 10.83 | 21.80 | 2.013 | 1E-18 | 14325 | Ftl1 | 327.68 | 1321.93 | 4.034 | 1E-19 |
| 69674 | Mif4gd | 4.78 | 9.98 | 2.087 | 2E-16 | 74617 | Scpep1 | 7.19 | 22.99 | 3.195 | 4E-21 | 13033 | Ctsd | 157.29 | 2962.84 | 18.837 | 1E-31 |
| 20416 | Shc1 | 3.43 | 10.07 | 2.940 | 9E-21 | 12193 | Zfp36l2 | 10.91 | 23.12 | 2.119 | 5E-17 | 11816 | Apoe | 1122.03 | 4869.31 | 4.340 | 5E-17 |

The levels of the genes in this cluster are increased by more than 2 folds with p-value < 0.05 at 3 days after stroke onset and peaked 4 days later (7 days after stroke).

© 2020. Wu J et al. Published online at http://www.aginganddisease.org/EN/10.14336/AD.2020.09206

**Supplementary Table 5.** A list of the DEGs in the cluster three.

| Gene ID | Gene | Sham | Stroke | Fold Change | p-value | Gene ID | Gene | Sham | Stroke | Fold Change | p-value |
|---|---|---|---|---|---|---|---|---|---|---|---|
| 56696 | Gpr132 | 0.004 | 0.51 | 130.848 | 2E-23 | 20969 | Sdc1 | 0.328 | 3.83 | 11.673 | 4E-38 |
| 56318 | Acpp | 0.066 | 0.51 | 7.750 | 9E-21 | 20311 | Cxcl5 | 0.221 | 3.91 | 17.635 | 1E-36 |
| 12399 | Runx3 | 0.005 | 0.51 | 109.729 | 5E-33 | 17474 | Clec4d | 0.009 | 4.14 | 443.457 | 9E-36 |
| 12766 | Cxcr3 | 0.048 | 0.60 | 12.422 | 3E-24 | 16197 | Il7r | 0.177 | 4.19 | 23.716 | 9E-33 |
| 14765 | Gpr50 | 0.198 | 0.62 | 3.134 | 8E-20 | 76681 | Trim12a | 1.211 | 4.36 | 3.604 | 9E-30 |
| 76074 | Gbp8 | 0.071 | 0.63 | 8.869 | 9E-30 | 19214 | Ptgdr | 0.317 | 4.38 | 13.844 | 8E-26 |
| 21939 | Cd40 | 0.138 | 0.63 | 4.597 | 4E-15 | 27047 | Omd | 1.034 | 4.54 | 4.385 | 6E-17 |
| 241452 | Dhrs9 | 0.032 | 0.68 | 21.469 | 4E-20 | 12362 | Casp1 | 1.685 | 4.62 | 2.744 | 4E-16 |
| 30925 | Slamf6 | 0.027 | 0.68 | 25.395 | 2E-25 | 11489 | Adam12 | 0.742 | 4.66 | 6.280 | 3E-14 |
| 12525 | Cd8a | 0.037 | 0.69 | 18.669 | 3E-42 | 433791 | Gm13251 | 2.213 | 4.89 | 2.209 | 5E-19 |
| 329436 | Gm14461 | 0.006 | 0.69 | 125.016 | 3E-44 | 12818 | Col14a1 | 0.358 | 4.94 | 13.829 | 9E-29 |
| 14945 | Gzmk | 0.056 | 0.70 | 12.449 | 2E-15 | 12984 | Csf2rb2 | 0.199 | 5.12 | 25.713 | 6E-81 |
| 22780 | Ikzf3 | 0.035 | 0.75 | 21.375 | 1E-31 | 15442 | Hpse | 0.444 | 5.17 | 11.653 | 2E-30 |
| 238393 | Serpina3f | 0.005 | 0.76 | 160.987 | 2E-18 | 73149 | Clec4a3 | 0.439 | 5.30 | 12.088 | 6E-22 |
| 170786 | Cd209a | 0.287 | 0.78 | 2.715 | 6E-16 | 212943 | Fam46a | 1.637 | 5.36 | 3.273 | 2E-27 |
| 327766 | Tmem26 | 0.117 | 0.82 | 7.012 | 5E-22 | 21922 | Clec3b | 1.281 | 5.42 | 4.230 | 5E-31 |
| 70433 | Draxin | 0.144 | 0.82 | 5.728 | 3E-26 | 63986 | Gmfg | 1.111 | 5.47 | 4.921 | 1E-27 |
| 74568 | Mlkl | 0.080 | 0.85 | 10.662 | 2E-25 | 14049 | Eya2 | 0.820 | 5.60 | 6.829 | 8E-27 |
| 209558 | Enpp3 | 0.193 | 0.86 | 4.469 | 1E-22 | 208164 | Fam180a | 1.223 | 5.60 | 4.578 | 2E-21 |
| 433801 | Gm13212 | 0.105 | 0.87 | 8.317 | 7E-21 | 16516 | Kcnj15 | 0.014 | 5.69 | 410.777 | 2E-38 |
| 54483 | Mefv | 0.003 | 0.91 | 304.338 | 5E-22 | 21928 | Tnfaip2 | 0.193 | 5.73 | 29.681 | 1E-26 |
| 18725 | Pira2 | 0.009 | 0.91 | 101.464 | 8E-42 | 18733 | Pirb | 0.081 | 5.79 | 71.471 | 2E-54 |
| 20343 | Sell | 0.056 | 0.97 | 17.144 | 3E-16 | 71934 | Car13 | 0.555 | 5.82 | 10.484 | 8E-29 |
| 21426 | Tfec | 0.031 | 0.97 | 31.133 | 2E-29 | 214642 | Cped1 | 1.514 | 5.89 | 3.890 | 7E-17 |
| 227929 | Cytip | 0.040 | 1.02 | 25.723 | 9E-44 | 16847 | Lepr | 1.482 | 5.93 | 4.003 | 7E-23 |
| 50701 | Elane | 0.025 | 1.07 | 42.418 | 4E-28 | 209086 | Samd9l | 2.158 | 6.11 | 2.832 | 5E-19 |
| 11630 | Aim1 | 0.307 | 1.11 | 3.599 | 3E-20 | 18214 | Ddr2 | 1.604 | 6.21 | 3.875 | 4E-20 |
| 20471 | Six1 | 0.099 | 1.12 | 11.319 | 5E-15 | 14727 | Lilr4b | 0.037 | 6.43 | 172.980 | 9E-59 |
| 17312 | Clec10a | 0.265 | 1.16 | 4.362 | 2E-14 | 12263 | C2 | 0.616 | 6.47 | 10.503 | 3E-39 |
| 16534 | Kcnn4 | 0.165 | 1.16 | 7.049 | 3E-20 | 11425 | Apoc4 | 0.000 | 6.67 | #VALUE! | 2E-14 |
| 16421 | Itgb7 | 0.118 | 1.17 | 9.925 | 1E-38 | 15229 | Foxd1 | 0.833 | 6.74 | 8.091 | 4E-17 |
| 12274 | C6 | 0.003 | 1.17 | 356.572 | 3E-67 | 208777 | Sned1 | 1.542 | 6.79 | 4.405 | 1E-23 |
| 15000 | H2-DMb2 | 0.311 | 1.19 | 3.815 | 1E-23 | 13587 | Ear2 | 0.162 | 6.85 | 42.171 | 9E-63 |
| 12481 | Cd2 | 0.052 | 1.19 | 23.091 | 1E-22 | 21826 | Thbs2 | 2.029 | 7.50 | 3.694 | 8E-19 |
| 20299 | Ccl22 | 0.069 | 1.25 | 18.080 | 2E-15 | 212898 | Dse | 2.079 | 7.79 | 3.747 | 4E-19 |
| 64817 | Svep1 | 0.343 | 1.25 | 3.643 | 1E-26 | 50908 | C1s1 | 0.384 | 7.81 | 20.355 | 7E-58 |
| 19260 | Ptpn22 | 0.199 | 1.33 | 6.687 | 6E-25 | 83675 | Bicc1 | 1.597 | 8.01 | 5.015 | 5E-22 |
| 100861753 | Gm21188 | 0.006 | 1.40 | 245.837 | 6E-23 | 11433 | Acp5 | 0.099 | 8.08 | 81.695 | 3E-43 |
| 74748 | Slamf8 | 0.062 | 1.42 | 22.906 | 9E-27 | 50501 | Prok2 | 0.345 | 8.19 | 23.770 | 2E-52 |
| 22403 | Wisp2 | 0.050 | 1.48 | 29.367 | 3E-34 | 117167 | Steap4 | 0.116 | 8.20 | 70.424 | 2E-51 |
| 12519 | Cd80 | 0.176 | 1.52 | 8.627 | 2E-19 | 13038 | Ctsk | 1.683 | 8.86 | 5.264 | 9E-21 |
| 665033 | Col6a5 | 0.130 | 1.52 | 11.716 | 5E-27 | 14863 | Gstm2 | 1.639 | 8.92 | 5.443 | 5E-18 |
| 83408 | Gimap3 | 0.055 | 1.62 | 29.353 | 1E-44 | 226101 | Myof | 1.080 | 8.96 | 8.293 | 5E-28 |
| 20715 | Serpina3g | 0.118 | 1.62 | 13.786 | 2E-21 | 14190 | Fgl2 | 0.903 | 9.15 | 10.132 | 9E-45 |
| 27052 | Aoah | 0.097 | 1.66 | 17.096 | 3E-47 | 16425 | Itih2 | 1.294 | 9.26 | 7.157 | 2E-26 |
| 246049 | Slc36a2 | 0.181 | 1.68 | 9.243 | 2E-40 | 12267 | C3ar1 | 1.440 | 9.63 | 6.688 | 1E-32 |
| 217304 | Cd300lb | 0.006 | 1.71 | 265.169 | 3E-51 | 50909 | C1ra | 0.856 | 9.91 | 11.588 | 5E-30 |
| 14058 | F10 | 0.017 | 1.84 | 106.668 | 3E-26 | 19264 | Ptprc | 0.795 | 9.94 | 12.503 | 9E-54 |
| 57781 | Cd200r1 | 0.323 | 1.89 | 5.846 | 3E-16 | 12494 | Cd38 | 1.389 | 10.06 | 7.240 | 3E-31 |
| 71712 | Dram1 | 0.404 | 1.92 | 4.770 | 9E-22 | 17300 | Foxc1 | 3.022 | 11.01 | 3.644 | 2E-17 |
| 22035 | Tnfsf10 | 0.762 | 2.13 | 2.798 | 1E-15 | 17381 | Mmp12 | 0.003 | 11.25 | 4450.032 | 1E-40 |
| 22420 | Wnt6 | 0.646 | 2.15 | 3.333 | 1E-15 | 12767 | Cxcr4 | 0.633 | 11.27 | 17.811 | 6E-39 |
| 18985 | Pou2af1 | 0.038 | 2.18 | 56.866 | 3E-30 | 16411 | Itgax | 0.054 | 11.46 | 213.512 | 2E-123 |

© 2020. Wu J et al. Published online at http://www.aginganddisease.org/EN/10.14336/AD.2020.09206

| | | | | | | | | | | | |
|---|---|---|---|---|---|---|---|---|---|---|---|
| 21384 | Tbx15 | 0.314 | 2.28 | 7.250 | 3E-14 | 12904 | Crabp2 | 2.293 | 11.94 | 5.210 | 3E-30 |
| 54199 | Ccrl2 | 0.307 | 2.31 | 7.533 | 8E-33 | 13058 | Cybb | 0.201 | 12.22 | 60.930 | 1E-48 |
| 12518 | Cd79a | 0.158 | 2.38 | 15.074 | 2E-44 | 12491 | Cd36 | 0.559 | 12.39 | 22.157 | 2E-14 |
| 100040462 | Mndal | 0.312 | 2.47 | 7.907 | 3E-29 | 68428 | Steap3 | 2.266 | 13.01 | 5.741 | 2E-28 |
| 269799 | Clec4a1 | 0.129 | 2.53 | 19.607 | 2E-24 | 69590 | Gpx8 | 3.271 | 13.35 | 4.083 | 2E-18 |
| 16175 | Il1a | 0.370 | 2.54 | 6.869 | 3E-28 | 56264 | Cpxm1 | 2.205 | 14.80 | 6.712 | 2E-27 |
| 13809 | Enpep | 0.827 | 2.57 | 3.111 | 1E-19 | 20568 | Slpi | 0.080 | 14.85 | 186.025 | 2E-33 |
| 55987 | Cpxm2 | 0.848 | 2.62 | 3.087 | 6E-24 | 71660 | Rarres2 | 5.736 | 15.84 | 2.761 | 1E-16 |
| 11695 | Alx4 | 0.476 | 2.64 | 5.553 | 6E-18 | 13078 | Cyp1b1 | 0.928 | 15.85 | 17.076 | 2E-32 |
| 17386 | Mmp13 | 0.021 | 2.69 | 127.604 | 1E-19 | 16790 | Anpep | 1.470 | 17.24 | 11.723 | 8E-36 |
| 60361 | Ms4a4b | 0.111 | 2.71 | 24.309 | 2E-52 | 11746 | Anxa4 | 1.913 | 17.73 | 9.267 | 7E-38 |
| 12363 | Casp4 | 0.238 | 2.72 | 11.417 | 7E-36 | 14619 | Gjb2 | 4.135 | 19.08 | 4.614 | 2E-18 |
| 22038 | Plscr1 | 0.860 | 2.76 | 3.211 | 2E-18 | 102680 | Slc6a20a | 6.387 | 19.43 | 3.043 | 6E-17 |
| 14191 | Fgr | 0.018 | 2.84 | 155.457 | 2E-47 | 140792 | Colec12 | 3.857 | 19.83 | 5.140 | 1E-30 |
| 107526 | Gimap4 | 0.129 | 2.85 | 22.180 | 1E-26 | 21824 | Thbd | 4.761 | 22.08 | 4.637 | 1E-16 |
| 63913 | Fam129a | 0.928 | 2.86 | 3.079 | 1E-17 | 242341 | Atp6v0d2 | 0.008 | 23.54 | 3124.263 | 1E-58 |
| 69816 | Mzb1 | 0.089 | 2.89 | 32.413 | 3E-48 | 20317 | Serpinf1 | 5.007 | 23.64 | 4.721 | 9E-23 |
| 242939 | Cpz | 0.074 | 2.94 | 39.837 | 1E-28 | 18295 | Ogn | 4.326 | 24.19 | 5.592 | 2E-22 |
| 16997 | Ltbp2 | 0.086 | 2.94 | 34.025 | 9E-42 | 11770 | Fabp4 | 0.135 | 25.62 | 189.367 | 2E-56 |
| 230979 | Tnfrsf14 | 0.462 | 3.05 | 6.603 | 7E-24 | 14114 | Fbln1 | 2.417 | 26.52 | 10.974 | 1E-29 |
| 12364 | Casp12 | 0.578 | 3.07 | 5.303 | 1E-25 | 16952 | Anxa1 | 1.101 | 26.81 | 24.354 | 1E-47 |
| 71724 | Aox3 | 0.558 | 3.08 | 5.526 | 1E-33 | 68713 | Ifitm1 | 2.542 | 28.57 | 11.239 | 4E-40 |
| 407800 | Ecm2 | 1.255 | 3.08 | 2.457 | 6E-14 | 56615 | Mgst1 | 7.246 | 28.71 | 3.963 | 6E-27 |
| 434484 | Sp140 | 0.338 | 3.13 | 9.252 | 2E-32 | 16956 | Lpl | 6.307 | 29.08 | 4.611 | 3E-18 |
| 75345 | Slamf7 | 0.013 | 3.13 | 242.607 | 7E-58 | 14710 | Gngt2 | 3.679 | 31.26 | 8.498 | 3E-44 |
| 56620 | Clec4n | 0.139 | 3.17 | 22.783 | 2E-23 | 14130 | Fcgr2b | 1.784 | 31.54 | 17.684 | 7E-75 |
| 22259 | Nr1h3 | 1.015 | 3.17 | 3.119 | 8E-23 | 17022 | Lum | 1.171 | 34.41 | 29.384 | 2E-34 |
| 66857 | Plbd1 | 0.076 | 3.21 | 42.242 | 4E-36 | 12870 | Cp | 4.614 | 36.42 | 7.894 | 6E-38 |
| 319236 | Trim12c | 0.925 | 3.27 | 3.536 | 1E-22 | 11568 | Aebp1 | 5.997 | 38.20 | 6.369 | 6E-24 |
| 71683 | Gypc | 0.688 | 3.35 | 4.875 | 2E-22 | 216616 | Efemp1 | 5.354 | 39.39 | 7.357 | 8E-29 |
| 170744 | Tlr8 | 0.075 | 3.36 | 44.790 | 2E-29 | 93694 | Clec2d | 11.016 | 41.51 | 3.768 | 2E-27 |
| 69772 | Bdh2 | 1.090 | 3.39 | 3.110 | 5E-21 | 14969 | H2-Eb1 | 1.020 | 41.53 | 40.714 | 1E-67 |
| 12772 | Ccr2 | 0.129 | 3.49 | 26.995 | 7E-18 | 56644 | Clec7a | 0.199 | 43.88 | 220.246 | 5E-57 |
| 12038 | Bche | 1.542 | 3.50 | 2.269 | 3E-16 | 20379 | Sfrp4 | 0.410 | 46.97 | 114.644 | 3E-46 |
| 11796 | Birc3 | 0.765 | 3.61 | 4.712 | 6E-27 | 12925 | Crip1 | 11.868 | 52.01 | 4.383 | 3E-19 |
| 71914 | Antxr2 | 0.652 | 3.62 | 5.547 | 3E-25 | 20305 | Ccl6 | 1.284 | 53.09 | 41.352 | 2E-91 |
| 76365 | Tbx18 | 0.794 | 3.68 | 4.627 | 8E-15 | 12258 | Serping1 | 3.072 | 55.19 | 17.967 | 1E-54 |
| 232413 | Clec12a | 0.321 | 3.75 | 11.680 | 1E-15 | 12266 | C3 | 0.146 | 66.76 | 455.886 | 7E-47 |
| 20204 | Prrx2 | 0.508 | 3.78 | 7.441 | 5E-26 | 14778 | Gpx3 | 6.985 | 67.61 | 9.680 | 7E-31 |
| 67606 | Fibin | 1.260 | 3.78 | 3.002 | 2E-18 | 11812 | Apoc1 | 2.798 | 67.82 | 24.238 | 1E-34 |
| 114774 | Pawr | 1.454 | 3.80 | 2.615 | 2E-17 | 80876 | Ifitm2 | 11.867 | 71.74 | 6.045 | 9E-26 |

The expression levels of the genes in this cluster are increased by more than 2 fold with p-value < 0.05 at 14 days after stroke onset and peaked 14 days later (28 days after stroke).

© 2020. Wu J et al. Published online at http://www.aginganddisease.org/EN/10.14336/AD.2020.09206

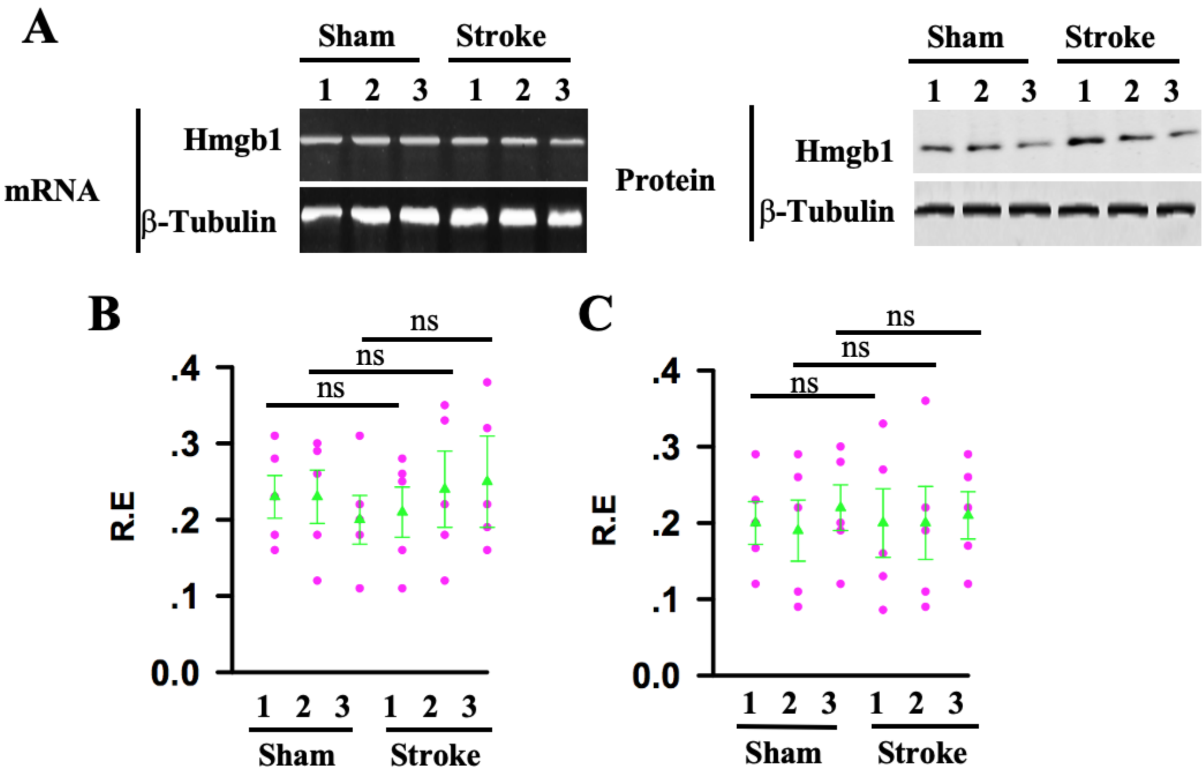


**Supplementary Figure 1. Stroke produces no changes in the expression of Hmgb1. A-C)** representative images (**A**) of Hmgb1 mRNA and protein in the microglia lysates from mice 1 (lane 1), 2 (lane 2), or 3 (lane 3) days after operation with sham or stroke. Relative expression (R.E) levels of mRNA (**B**) and protein (**C**), defined by normalizing the band intensities of Hmgb1 blots to the respective a-tubulin) in the individual mice (circles) and their averages per group (triangles) are plotted. Data are mean ± SEM (n = 5 mice per group, ns = no significantly differences, *t*-tests).

© 2020. Wu J et al. Published online at http://www.aginganddisease.org/EN/10.14336/AD.2020.09206

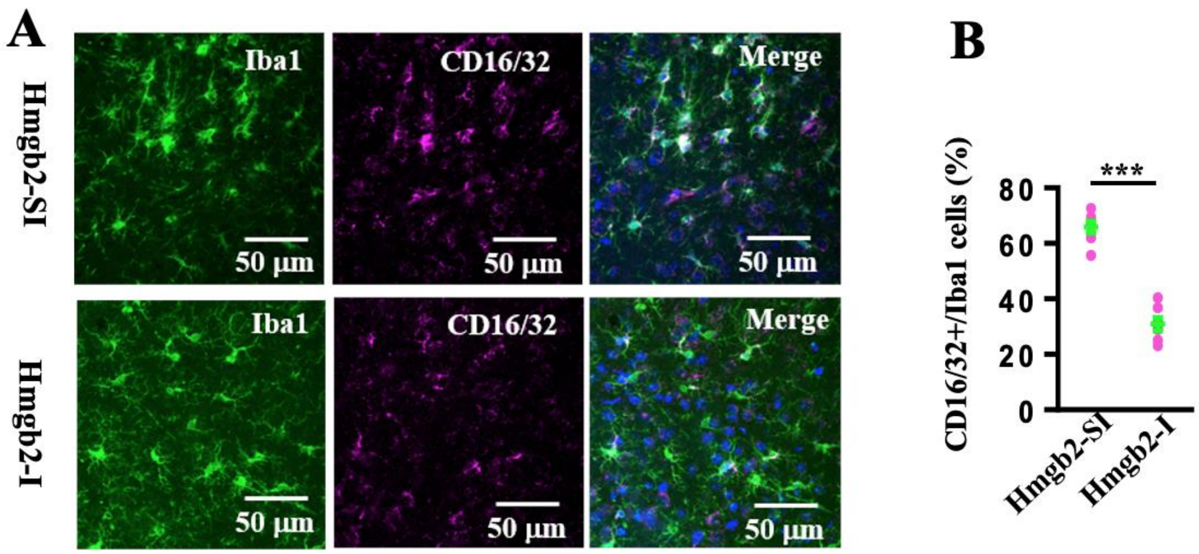


**Supplementary Figure 2. Knocking down Hmgb2 in microglia inhibited microglia pro-inflammatory response. (A)** representative images show the expression of CD16/32+ (M1 marker, purple) and Iba1 (green) in the cortex at 3days after the operation which operated at 18 days after the injection of the AAV-PHP.eB-Hmgb2-SI/tdT or the AAV-PHP.eB-DIO-Hmgb2-I/tdT virus particles into the tail vein of the Cx3cr1-Cre mice. **(B)** quantification of the percentage of CD16/32+/Iba1+ cells are analyzed and plotted by the individual sections (circles) and their averages per group (triangles). Data are mean ± SEM(n=7 per group, ***p <0.0001, ns = no significantly differences)

© 2020. Wu J et al. Published online at http://www.aginganddisease.org/EN/10.14336/AD.2020.09206

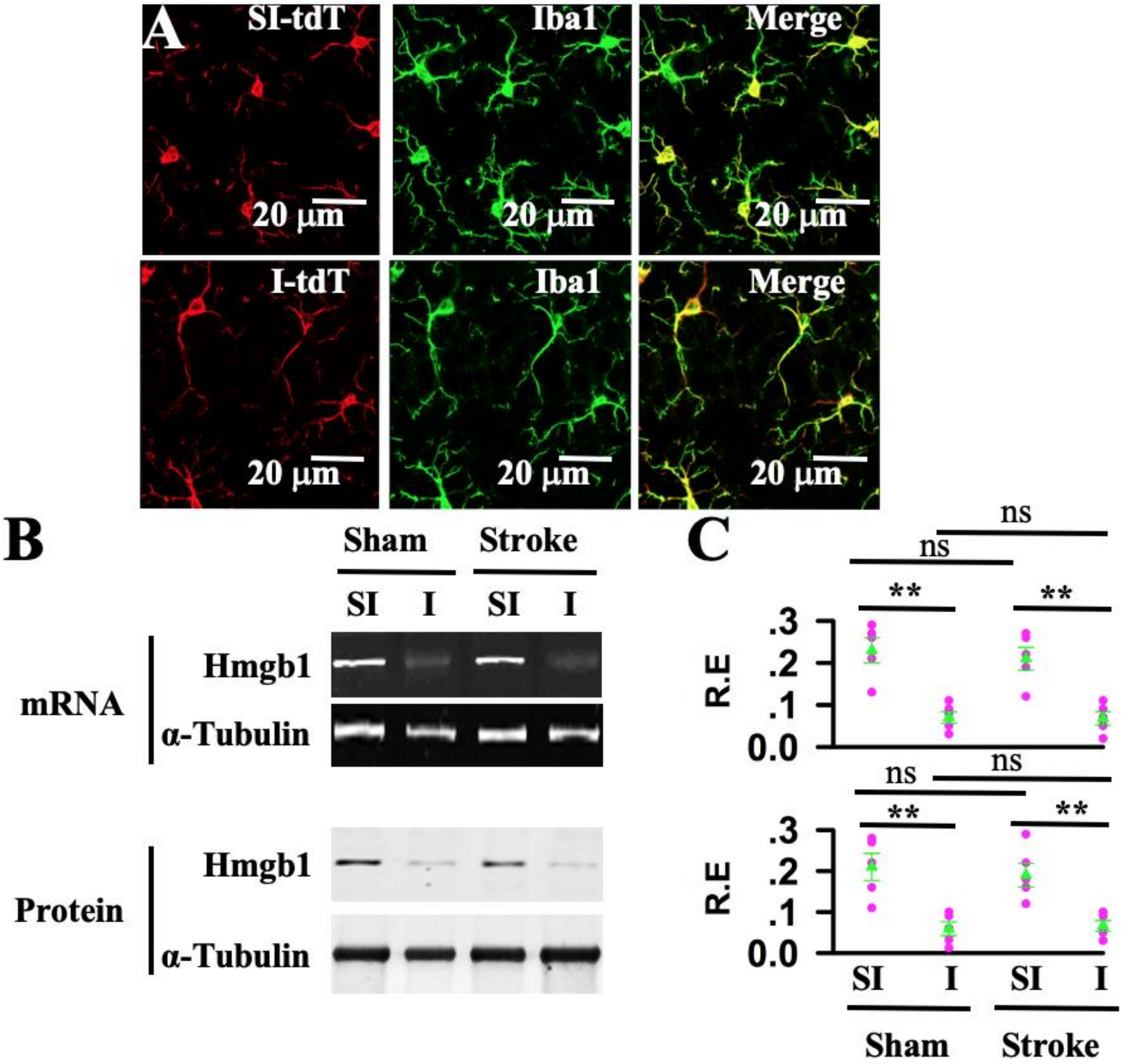


**Supplementary Figure 3**. **Knocking down Hmgb1 in microglia. (A)** representative images show the expression of Hmgb1-SI (red) or Hmgb1-I (red) in microglia (green) 18 days after the injection of a high titer of the AAV-PHP.eB-DIO-Hmgb1-SI/tdT or the AAV-PHP.eB-DIO-Hmgb1-I/tdT virus particles into the tail vein of Cx3cr1-Cre mice. **(B), (C),** representatives **(B)** of Hmgb1 mRNA and protein in the microglia lysates from mice with the expression of Hmgb1-SI or Hmgb1-I 3 days after operation with sham or stroke. Relative expression (R.E) levels of mRNA **(C, top)** and protein **(C, bottom)** by normalizing the band intensities of Hmgb1 blots to the respective β-tubulin) in the individual mice (circles) and their averages per group (triangles) are plotted. Data are mean ± SEM (n = 5 mice per group, ns = no significantly differences, **p = 0.0011, 0.0017, 0.0035, 0.0037, t-tests).

© 2020. Wu J et al. Published online at http://www.aginganddisease.org/EN/10.14336/AD.2020.09206

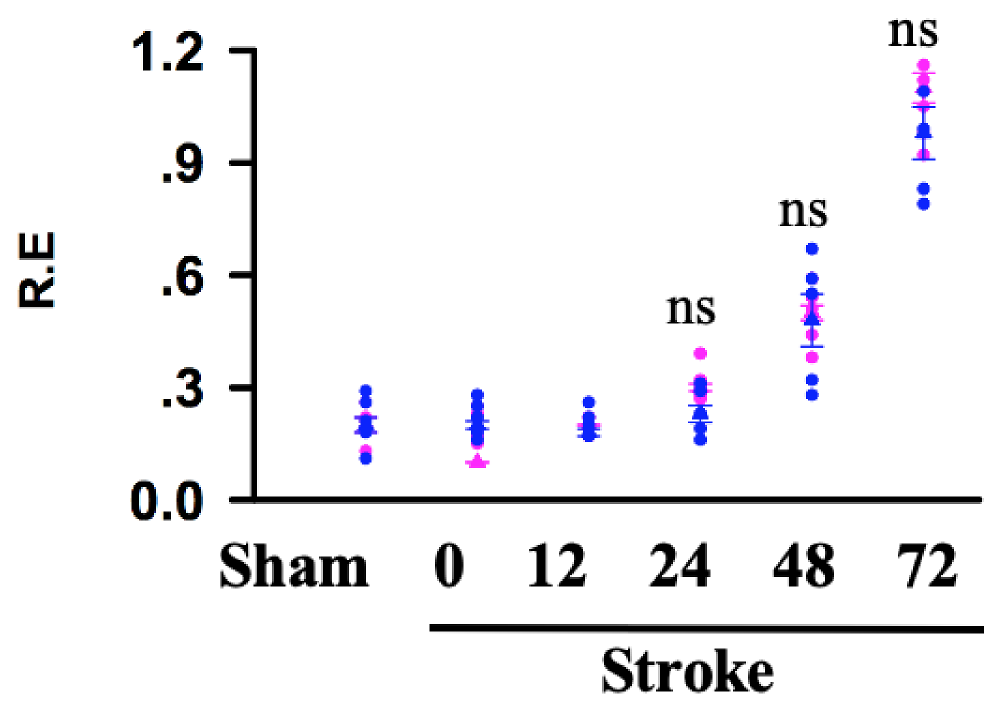


**Supplementary Figure 4. Hmgb1-I does not alter the expression of Hmgb2 in microglia.** Mice with the expression of Hmgb1-I (blue symbols) or Hmgb1-SI (pink symbols) were operated with sham or stroke. 0, 12, 24, 48, or 72 hours after the operation, microglia lysates were prepared and blotted with anti-Hmgb2 or anti-a-tubulin. Relative expression (R.E) levels (defined by normalizing the band intensities of anti-Hmgb2 to the respective a-tubulin) in the individual mice (circles) and their averages per group are plotted. Data are mean ± SEM ( n = 5 mice per group, ns = no significantly differences, between Hmgb1-I and Hmgb1-SI, t-tests).

© 2020. Wu J et al. Published online at http://www.aginganddisease.org/EN/10.14336/AD.2020.09206

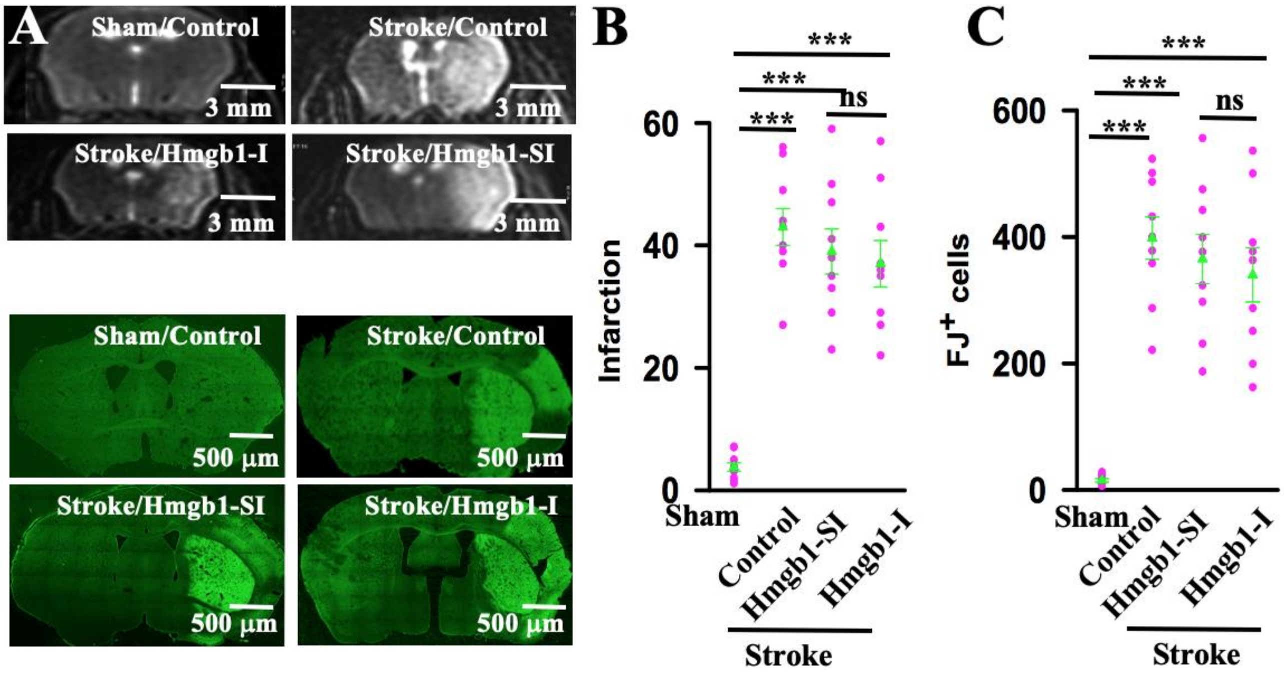


**Supplementary Figure 5. Inhibition of Hmgb1 does not protect stroke damages**. **(A)** representative T2 weighted MR images (top) and FJ labeling (bottom) from sham and stroke mice without (control) or with the expression of Hmgb1-SI or Hmgb1-I. **(B), (C),** the infarction (B, mean ± SEM, n = 9 mice per group, $F(3, 32) = 35.44$, ns = no significantly differences, ***$p < 0.0001$; BF ANOVA) and the FJ+ cells (C, mean ± SEM, n = 9 mice per group, $F(3, 32) = 28.17$, ns = no significantly differences, ***$p < 0.0001$; BF ANOVA) in the individual mice (circles) and the averages per group (triangles) are plotted.

© 2020. Wu J et al. Published online at http://www.aginganddisease.org/EN/10.14336/AD.2020.09206
